# Covariation of the Fecal Microbiome with Diet in Nonpasserine Birds

**DOI:** 10.1128/mSphere.00308-21

**Published:** 2021-05-12

**Authors:** Kangpeng Xiao, Yutan Fan, Zhipeng Zhang, Xuejuan Shen, Xiaobing Li, Xianghui Liang, Ran Bi, Yajiang Wu, Junqiong Zhai, Junwei Dai, David M. Irwin, Wu Chen, Yongyi Shen

**Affiliations:** a Guangdong Laboratory for Lingnan Modern Agriculture, Guangzhou, China; b College of Veterinary Medicine, South China Agricultural University, Guangzhou, China; c Guangzhou Zoo, Guangzhou, China; d Department of Laboratory Medicine and Pathobiology, University of Toronto, Toronto, Canada; e Banting and Best Diabetes Centre, University of Toronto, Toronto, Canada; f Key Laboratory of Zoonosis Prevention and Control of Guangdong Province, Guangzhou, China; Nanjing Normal University

**Keywords:** 16S rRNA, bird, diet, diet-microbiome-host, gut microbiome, metagenome

## Abstract

Opportunistic feeding and multiple other environment factors can modulate the gut microbiome, and bias conclusions, when wild animals are used for studying the influence of phylogeny and diet on their gut microbiomes. Here, we controlled for these other confounding factors in our investigation of the magnitude of the effect of diet on the gut microbiome assemblies of nonpasserine birds. We collected fecal samples, at one point in time, from 35 species of birds in a single zoo as well as 6 species of domestic poultry from farms in Guangzhou city to minimize the influences from interfering factors. Specifically, we describe 16S rRNA amplicon data from 129 fecal samples obtained from 41 species of birds, with additional shotgun metagenomic sequencing data generated from 16 of these individuals. Our data show that diets containing native starch increase the abundance of *Lactobacillus* in the gut microbiome, while those containing plant-derived fiber mainly enrich the level of *Clostridium*. Greater numbers of *Fusobacteria* and *Proteobacteria* are detected in carnivorous birds, while in birds fed a commercial corn-soybean basal diet, a stronger inner-connected microbial community containing *Clostridia* and *Bacteroidia* was enriched. Furthermore, the metagenome functions of the microbes (such as lipid metabolism and amino acid synthesis) were adapted to the different food types to achieve a beneficial state for the host. In conclusion, the covariation of diet and gut microbiome identified in our study demonstrates a modulation of the gut microbiome by dietary diversity and helps us better understand how birds live based on diet-microbiome-host interactions.

**IMPORTANCE** Our study identified food source, rather than host phylogeny, as the main factor modulating the gut microbiome diversity of nonpasserine birds, after minimizing the effects of other complex interfering factors such as weather, season, and geography. Adaptive evolution of microbes to food types formed a dietary-microbiome-host interaction reciprocal state. The covariation of diet and gut microbiome, including the response of microbiota assembly to diet in structure and function, is important for health and nutrition in animals. Our findings help resolve the major modulators of gut microbiome diversity in nonpasserine birds, which had not previously been well studied. The diet-microbe interactions and cooccurrence patterns identified in our study may be of special interest for future health assessment and conservation in birds.

## Abstract (precis)

Our study identified food source, rather than host phylogeny, as the main factor modulating the gut microbiome diversity of nonpasserine birds, after minimizing the effects of other complex interfering factors such as weather, season, and geography. Adaptive evolution of microbes to food types formed a dietary-microbiome-host interaction reciprocal state.

## INTRODUCTION

Digestive tracts of animals contain microbial communities that are composed of different bacterial groups with various abundances and functional characteristics (1). In addition to digestion and energy acquisition, many recent studies have shown that the animal gut microbiome also has important functions in host immune responses, detoxification, and behavior (2, 3). The adaptive capacity and health status of an animal are not solely due to the host genome but also depend upon the vast genetic repertoire of its microbiome (4).

Birds exhibit the most diverse range of ecological functions among vertebrates (5) and represent a highly evolved lineage that provides processes that are essential for ecological communities and agricultural ecosystems (6, 7). Coevolution between host and microbial lineages played key roles in the adaptation of mammals to their diverse lifestyles, but this subject has been far less studied in birds (8–11). Birds represent multiple different feeding groups including folivorous, nectar feeding, opportunistic, strictly carrion feeding, and others (12). Exploitation of a new dietary niche is a powerful driver for changes in gut microorganisms and their coevolution with their animal host (13). Many studies have shown the important effects of diet on birds, especially in *Passeriformes*, which represent more than half of all known species of birds (14–17). To date, however, few studies have characterized the gut microbiomes of nonpasserine birds and their associations with their highly diverse dietary habits.

The dynamic gut microecological system that formed in the adaption of birds to their environment is influenced by many factors, such as sex, reproductive status, age, geography, environment, human activity, and social structure (9, 18–23). The dominant drivers of gut microbiome diversity in birds appear to be host evolutionary history and diet (10). Diet drives not only taxonomic diversity but also the functional content of the gut microbiome in mammals (24, 25). While there have been many analyses of microbial taxa based on amplicon rRNA sequencing, fewer studies have focused on functional gut metagenome profiling in the dietary diversity of birds (26). Moreover, it is difficult to address questions on the identity of host-specific microbes, as the microbial species were often collected in different habitat niches and environmental conditions (e.g., weather and season) that have roles in influencing the composition of the gut microbiota (27). Therefore, to better reveal the complex relationship between diet and the gut microbiome in birds, it is necessary to minimize the influence of any interfering factors.

In this study, fecal material was collected from birds housed at Guangzhou Zoo representing 35 species with various dietary habits at one point in time to minimize the influence of external factors such as geography, weather, and season. In addition, we also compared the gut microbiota from 6 species of domestic poultry that were fed different types of food to identify differences in their gut microbiota that can occur in a species as a response to different food types. To understand the dietary and phylogenetic effects on the taxonomic composition and metabolic function of gut microbiota in birds, a systematic analysis combining 16S rRNA amplification and metagenomic sequencing was used.

## RESULTS

### Gut microbial diversity of nonpasserine birds.

**FIG 1:**
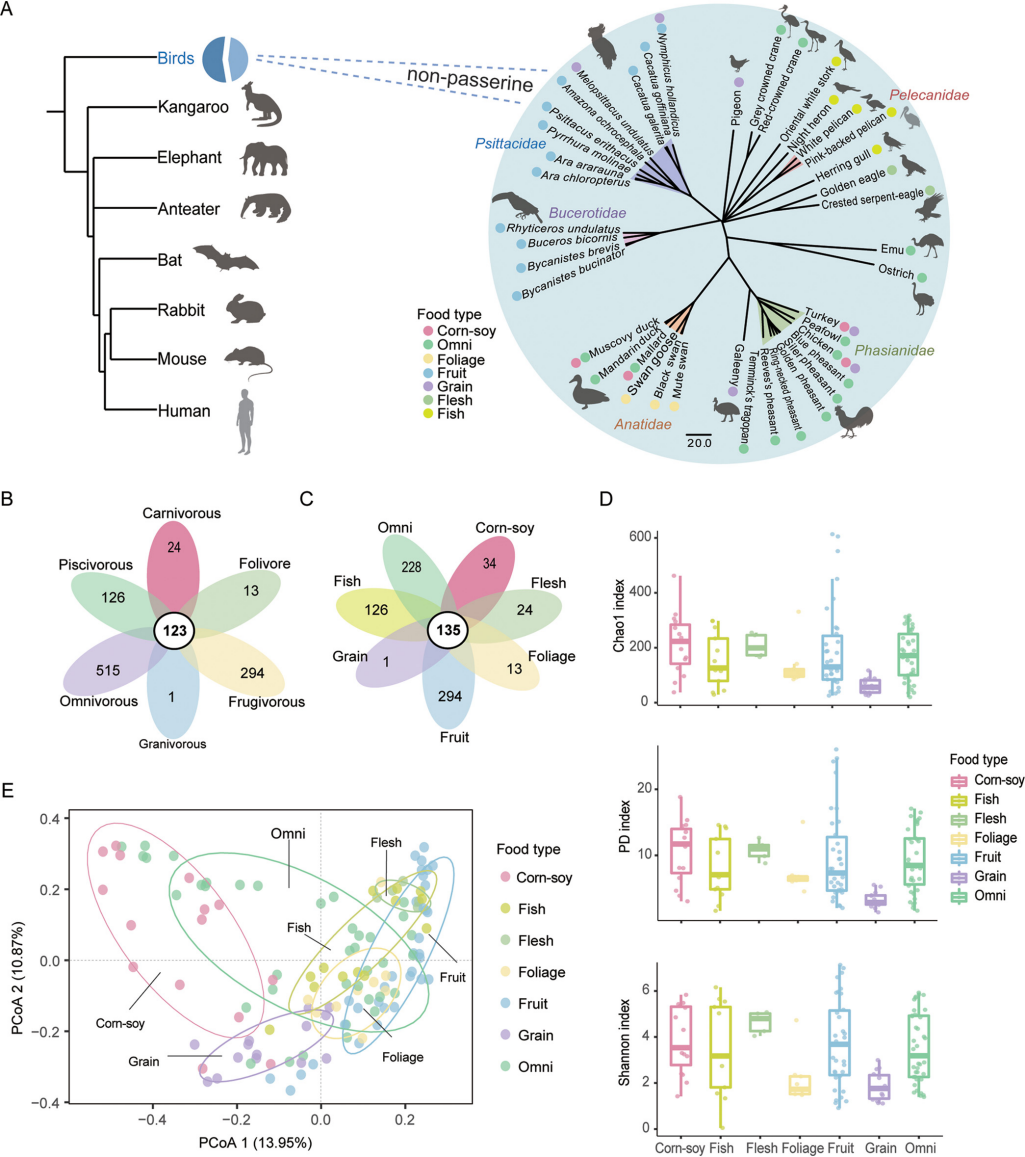
Diet type influences microbial diversity. (A) Phylogenetic tree of the birds used in this study. (B) Flower plot shows shared and unique OTUs between the 6 feeding habit groups. (C) Flower plot shows shared and unique OTUs between 7 dietary type groups. (D) Differences in microbial diversity (Chao1, PD whole tree, and Shannon index) at the OTU level between 7 dietary type groups shown as box plots (*t* test). (E) PCoA plot based on the Bray-Curtis dissimilarities at the OTU level. Differences observed between the groups are based on the PERMANOVA test. Results show that dietary type is a predictor of microbial variance (*r*^2^ = 0.21304, *P* = 0.0001). Each color corresponds to a dietary type. Ellipses are at the 70% confidence level.

To assess microbial diversity, we sequenced the V3-V4 regions of the 16S rRNA gene in 129 fecal samples from 41 species (classified in the orders *Gruiformes*, *Psittaciformes*, *Anseriformes*, *Accipitriformes*, *Galliformes*, *Pelecaniformes*, *Ciconiiformes*, *Bucerotiformes*, *Struthioniformes*, *Casuariiformes*, *Columbiformes*, and *Charadriiformes*) (Fig. 1A and see Table S1 in the supplemental material). In total, 128 samples passed our quality control process and 2,391 operational taxonomic units (OTUs) were identified. We first assessed the impact of general feeding habits on gut microbiome diversity, noting, however, that the food types of omnivores vary widely (Fig. 1B and Fig. S1). When we grouped according to seven classes of food types (fruits, corn-soy, grains, foliage, flesh, fish, and omnivore), we found that 135 OTUs were shared by all groups. The food type with the highest number of unique OTUs was the fruit food group (294 OTUs), followed by the omnivore group (228 OTUs) (Fig. 1C). In contrast, the fewest unique OTUs were detected in the grain (1 OTU), foliage (13 OTUs), and corn-soy (34 OTUs) food groups. Moreover, most OTUs (90%) were detected only in fewer than 20% of samples (Fig. S2).

10.1128/mSphere.00308-21.1 FIG S1 Feeding habits influence microbial diversity. (A) Venn diagram of the OTUs shared between the omnivorous, herbivorous, and carnivorous groups. (B) Differences in microbial diversity (Chao1, PD whole tree, and Shannon index) at the OTU level between the 6 feeding habit groups are shown as box plots. (C) PCoA plot based on the Bray-Curtis dissimilarities at the OTU level. Download FIG S1, TIF file, 1.0 MB. Copyright © 2021 Xiao et al. 2021 Xiao et al. https://creativecommons.org/licenses/by/4.0/ This content is distributed under the terms of the Creative Commons Attribution 4.0 International license.

10.1128/mSphere.00308-21.2 FIG S2 OTUs (A) and microbiota genera (B) are sparsely distributed in the data set. The prevalence of OTU or genera across all samples was calculated (found in at least one sample). Download FIG S2, TIF file, 0.9 MB. Copyright © 2021 Xiao et al. 2021 Xiao et al. https://creativecommons.org/licenses/by/4.0/ This content is distributed under the terms of the Creative Commons Attribution 4.0 International license.

10.1128/mSphere.00308-21.6 TABLE S1 Metadata for each sample used in this study. Download Table S1, DOCX file, 0.02 MB. Copyright © 2021 Xiao et al. 2021 Xiao et al. https://creativecommons.org/licenses/by/4.0/ This content is distributed under the terms of the Creative Commons Attribution 4.0 International license.

Next, we used Chao1, phylogenetic diversity (PD whole tree index), and Shannon index to illustrate bacterial richness and diversity within the communities based on OTUs level. The α-diversity index of the grain food group was the lowest, and significantly lower than the fruit, corn-soy, flesh, and omnivore groups with the Chao1, PD, or Shannon index (*t* test, *P* < 0.05). No significant differences were observed among the other groups (Fig. 1D and Table S2). A principal-coordinate analysis (PCoA) based on the Bray-Curtis dissimilarity values was used to assess the differences in bacterial community structure between the samples. The results of this analysis revealed a significant clustering of gut microbiota by diet groups (permutational multivariate analysis of variances [PERMANOVA], *P* < 0.001), with food types separating the microbial communities along the first principal coordinate (PC1, 13.95% of variance) (Fig. 1E).

10.1128/mSphere.00308-21.7 TABLE S2 Comparison of the α-diversity (Chao1 index, PD whole tree index, and Shannon index) between the pairwise groups. Download Table S2, DOCX file, 0.02 MB. Copyright © 2021 Xiao et al. 2021 Xiao et al. https://creativecommons.org/licenses/by/4.0/ This content is distributed under the terms of the Creative Commons Attribution 4.0 International license.

Based on the Mantel test, both host diet (*r* = 0.1623, *P* value = 0.0001) and phylogeny (*r* = 0.09667, *P* value = 0.0001) affect the gut microbiota composition of birds, but the influence of diet seems to be greater. To further assess the effects of host phylogeny and diet on the gut microbiome, we then compared the host phylogenetic tree with a Bray-Curtis dissimilarity-based UPGMA (unweighted pair group method with arithmetic mean) tree of the gut microbiota, with the results showing that the gut microbiomes of the different bird species mainly clustered based on food type (Fig. S3). Moreover, gut microbiota from domestic poultry species fed with different food types (e.g., Gallus gallus, Meleagris gallopavo, Anas platyrhynchos, and Cairina moschata) was diverse, and also mainly clustered according to their food type (Fig. S3). MaAsLin2 analysis (adjusted *P* value < 0.05) further revealed that diet plays a major role and accounts for 89.1% of the microbiota features (Fig. S4).

10.1128/mSphere.00308-21.3 FIG S3 Effects of host phylogeny on gut microbiome diversity. The host phylogenetic tree is shown on the left. UPGMA tree clustering based on Bray-Curtis dissimilarities of gut microbiome is on the right. Sample and host are connected by the straight lines. Download FIG S3, JPG file, 1.9 MB. Copyright © 2021 Xiao et al. 2021 Xiao et al. https://creativecommons.org/licenses/by/4.0/ This content is distributed under the terms of the Creative Commons Attribution 4.0 International license.

10.1128/mSphere.00308-21.4 FIG S4 Effects of diet and phylogeny on the gut microbiome based on the MaAsLin2 analysis. (A) Pie chart of the significantly explained gut microbiome by diet and phylogeny. (B) Box plots show the BH-adjusted *P* values. Download FIG S4, TIF file, 0.4 MB. Copyright © 2021 Xiao et al. 2021 Xiao et al. https://creativecommons.org/licenses/by/4.0/ This content is distributed under the terms of the Creative Commons Attribution 4.0 International license.

### Dietary diversity affects predominant bacteria.

**FIG 2:**
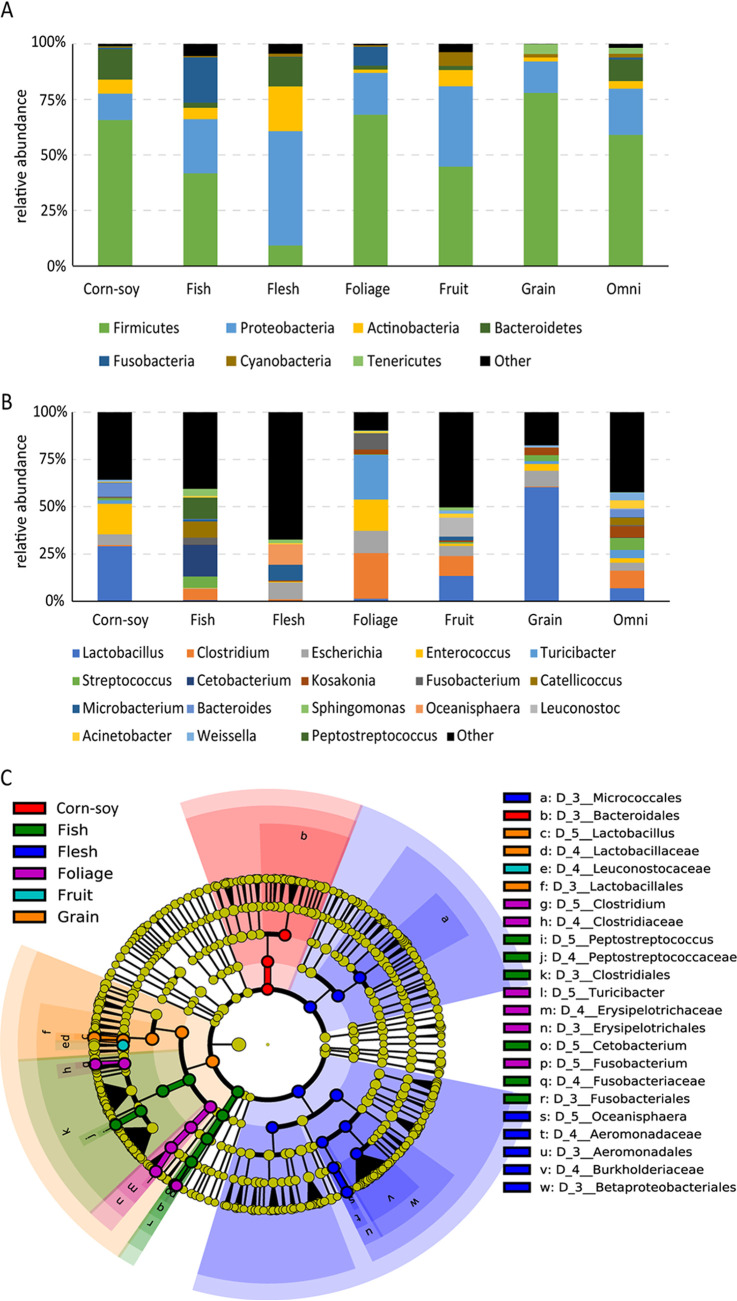
Microbial composition in the different dietary groups. (A and B) The bar plot shows taxa with average relative abundances higher than 1% at the phylum level (A) and the genus level (B). Remaining species are classified as other. (C) LEfSe analysis. Cladogram showing the differences in relative abundance of taxa at five levels between the 7 dietary groups. Plot showing the taxonomic levels represented by rings with phyla in the outermost ring and genera in the innermost ring. Circles with nonyellow color indicate that there is a significant difference in the relative abundance at the different taxon levels (Wilcoxon rank sum test, *P* < 0.01; LDA score > 4), and yellow circles indicate nonsignificant differences.

OTUs and genera were sparsely distributed in all samples (Fig. S2). However, the predominant bacterial phyla present in the feces of all birds were *Firmicutes* (mean abundance ranged from 9.19% to 76.49%), *Proteobacteria* (11.14% to 51.48%), *Actinobacteria* (1.53% to 20.22%), and *Bacteroidetes* (0.02% to 13.78%) (Fig. 2A). Notably, the most abundant phylum in the flesh-eating birds was *Proteobacteria*, while *Firmicutes* was the most abundant in the others. At the genus level, the five most abundant genera were *Lactobacillus*, *Clostridium*, *Enterococcus*, Escherichia, and *Turicibacter* (Fig. 2B). Consistent with the α-diversity characteristic, the number of genera with mean relative abundance of >1% in the omnivore food group was higher than in the other groups.

Differences in the abundance of the bacterial taxa were determined through a linear discriminant analysis effect size (LEfSe) analysis. Differentially abundant taxa were considered when there was significant variation between any two groups. A total of 35 taxa, at different classification levels, were found to have significant differences (Wilcoxon rank sum test, *P* < 0.01) (Fig. 2C). At the genus level, *Lactobacillus*, *Clostridium*, *Oceanisphaera*, and *Cetobacterium* were the dominant genera and were significantly more abundant in the grain, foliage, flesh, and fish food groups, respectively (Wilcoxon rank sum test, *P* < 0.01). At the order level, a significant enrichment of *Bacteroidales* was detected in the corn-soy food group. No significantly enriched taxa were observed in the omnivore food group.

### Microbial cooccurrence association patterns are influenced by diet.

**FIG 3:**
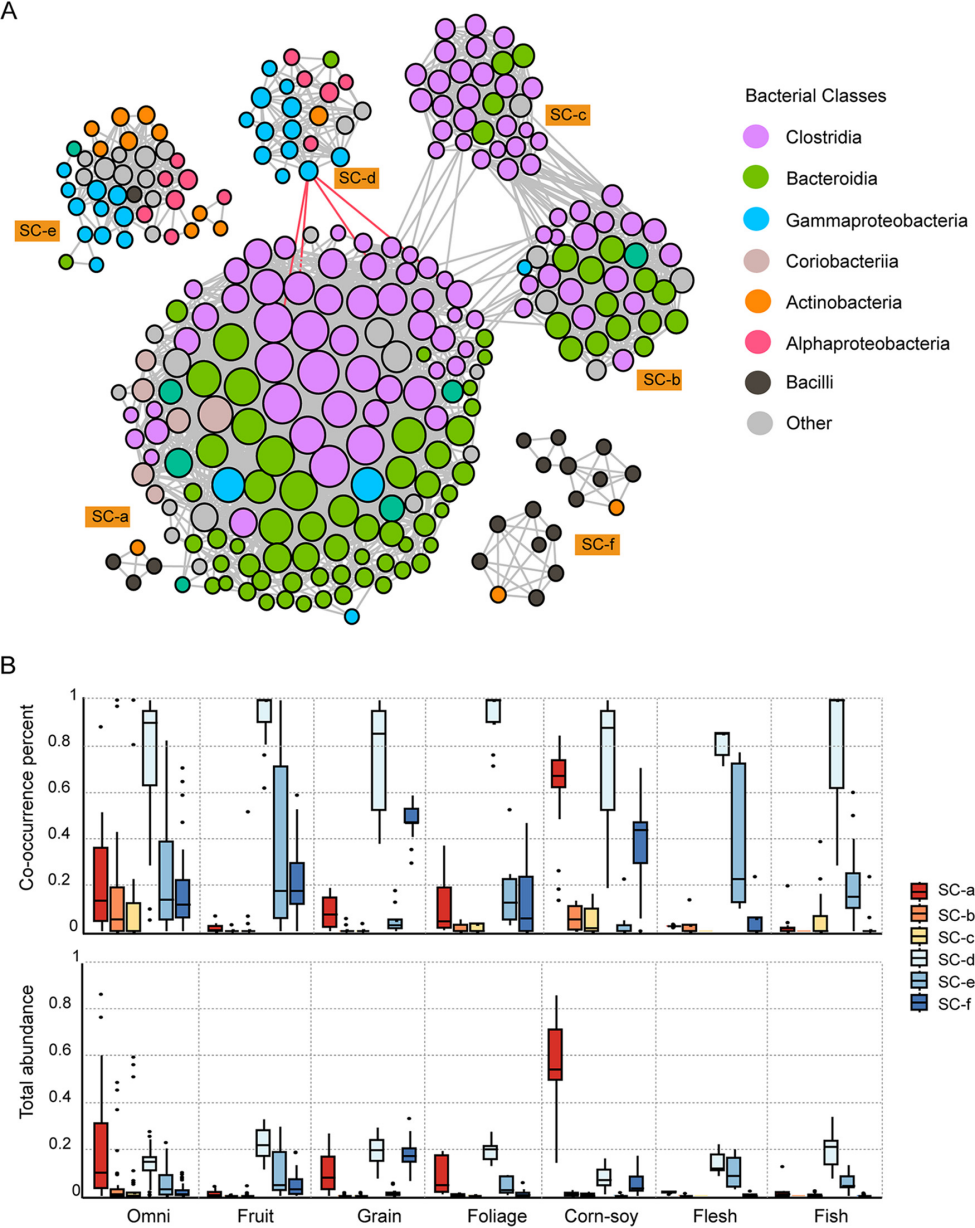
Microbial community linkages and species coexistence in the gut microbiome of birds. (A) Colored cooccurrence network of microbial taxa. Each node represents one OTU, and each edge represents a strong (|ρ| > 0.6) and significant correlation (FDR *P* < 0.01) between the two nodes. The size of each node is proportional to the degree of the OTUs; the thickness of edges is proportional to the value of the Spearman correlation coefficient. Gray edge, positive correlation (ρ > 0.6); red edge, negative correlation (ρ < −0.6). (B) Box plot of the completeness and richness of each submicrobial group (SC) in the different dietary groups. Cooccurrence percentage represents the completeness, and total abundance represents the richness.

We next examined how bacterial species cooccur among the birds in our study, which might be due to dietary differences or microbe-microbe interactions. A network containing 285 nodes and 2,689 edges was constructed (Fig. 3A and Table S3). Based on the layout structure, this integrated network could be divided into 6 subnetworks, each with differing taxonomic compositions at the class level (Fig. 3A and Table S4).

10.1128/mSphere.00308-21.8 TABLE S3 List of a set of topological metrics of the cooccurrence network. Download Table S3, DOCX file, 0.01 MB. Copyright © 2021 Xiao et al. 2021 Xiao et al. https://creativecommons.org/licenses/by/4.0/ This content is distributed under the terms of the Creative Commons Attribution 4.0 International license.

10.1128/mSphere.00308-21.9 TABLE S4 Taxonomic annotation of the OTUs in each subnetwork. Download Table S4, DOCX file, 0.1 MB. Copyright © 2021 Xiao et al. 2021 Xiao et al. https://creativecommons.org/licenses/by/4.0/ This content is distributed under the terms of the Creative Commons Attribution 4.0 International license.

At the class level, the cooccurrence network was mainly composed of interactions of *Clostridia*, *Bacteroidia*, *Gammaproteobacteria*, and *Bacilli*. Subcommunity a (SC-a), subcommunity b (SC-b), and subcommunity c (SC-c) were dominated by *Clostridia* and *Bacteroidia*; subcommunity d (SC-d) and subcommunity e (SC-e) possessed more members of *Actinobacteria*, *Gammaproteobacteria*, and *Alphaproteobacteria*; SC-f was mostly composed of taxa from *Bacilli.* In addition, most of the interactions were positive, while negative interactions appeared only between Pseudomonas (classified in *Gammaproteobacteria*) in SC-d with *Negativibacillus*, *Ruminococcaceae UCG-014*, *Intestinimonas,* and *Christensenellaceae R-7 group* (classified in *Clostridia*) in SC-a (Fig. 3A), suggesting that competitive inhibition occurs between these communities.

We then calculated the cooccurrence percentage and total abundance of each submicrobial community to estimate the microbial coexistence in the different diet groups. The presence and abundance of OTUs from each subnetwork differed substantially among the groups (Fig. 3B). SC-d was generally the most prevalent in all birds, while wide differences in the prevalence and abundance of SC-a and SC-f occurred among the groups, suggesting certain host dietary specificity of these microbial consortia. The abundance of SC-a in the corn-soy group was significantly higher than in the other groups, while the abundance of SC-d in the corn-soy group was the lowest among all groups (Wilcoxon rank sum test, *P* < 0.05) (Table S5). This phenomenon is consistent with antagonism between SC-a and SC-d as described above. We also compared the gut microbiome of some birds in our study with those previously reported from wild birds (*Cairina moschata* and Dromaius novaehollandiae) (8, 10). A greater abundance of *Proteobacteria* was observed in the wild birds (data not shown). Compared with birds fed commercial feed, there were more Gram-negative bacteria in the gut microbiome of zoo birds and wild birds, which may be related to their complex food types and environment.

10.1128/mSphere.00308-21.10 TABLE S5 Comparison of the cooccurrence percent and total abundance of the microbiota communities between the pairwise groups. BH-adjusted *P* values of <0.05 are colored (red, percent; green, abundance). Download Table S5, DOCX file, 0.03 MB. Copyright © 2021 Xiao et al. 2021 Xiao et al. https://creativecommons.org/licenses/by/4.0/ This content is distributed under the terms of the Creative Commons Attribution 4.0 International license.

### Adaptive evolution of microbial functions to fit food types.

To further investigate the functional capacities of the gut microbial communities in birds, a metagenomic analysis was conducted. The 16 samples used for the metagenomic analysis are distributed across the 6 food type groups (corn-soy, flesh, foliage, fruit, grain, and “omni”). In total, 2,420,003 assembled genes (92.3%, 2,621,823) were identified from the prokaryotic microbes and fungi by searches against the NCBI NR database. Of this total, 1,729,192 (65.95%) and 52,051 (1.99%) were annotated in the KEGG and CAZy (carbohydrate-active enzyme) databases, respectively.

Detailed KEGG orthology (KO) annotation information is listed in Fig. S5A. Notably, 2,508 (28.47%) of the KOs were annotated in the global and overview metabolism pathways and 741 KOs were annotated in carbohydrate metabolism. The number of KOs with an average relative abundance higher than 0.01% in the different groups ranged from 1,329 to 2,779, and the total abundance of those KOs in each group was higher than 85% (Fig. S5B). This indicates that the high-abundance KOs cover most of the microbial functions.

10.1128/mSphere.00308-21.5 FIG S5 Functional annotation of gut microbiome of nonpasserine birds. (A) Pathway classification of all annotated KOs in the metagenomic sequencing. (B) Count and total abundance of KOs with relative abundance higher than 0.01% in each group. Download FIG S5, TIF file, 1.9 MB. Copyright © 2021 Xiao et al. 2021 Xiao et al. https://creativecommons.org/licenses/by/4.0/ This content is distributed under the terms of the Creative Commons Attribution 4.0 International license.

**FIG 4:**
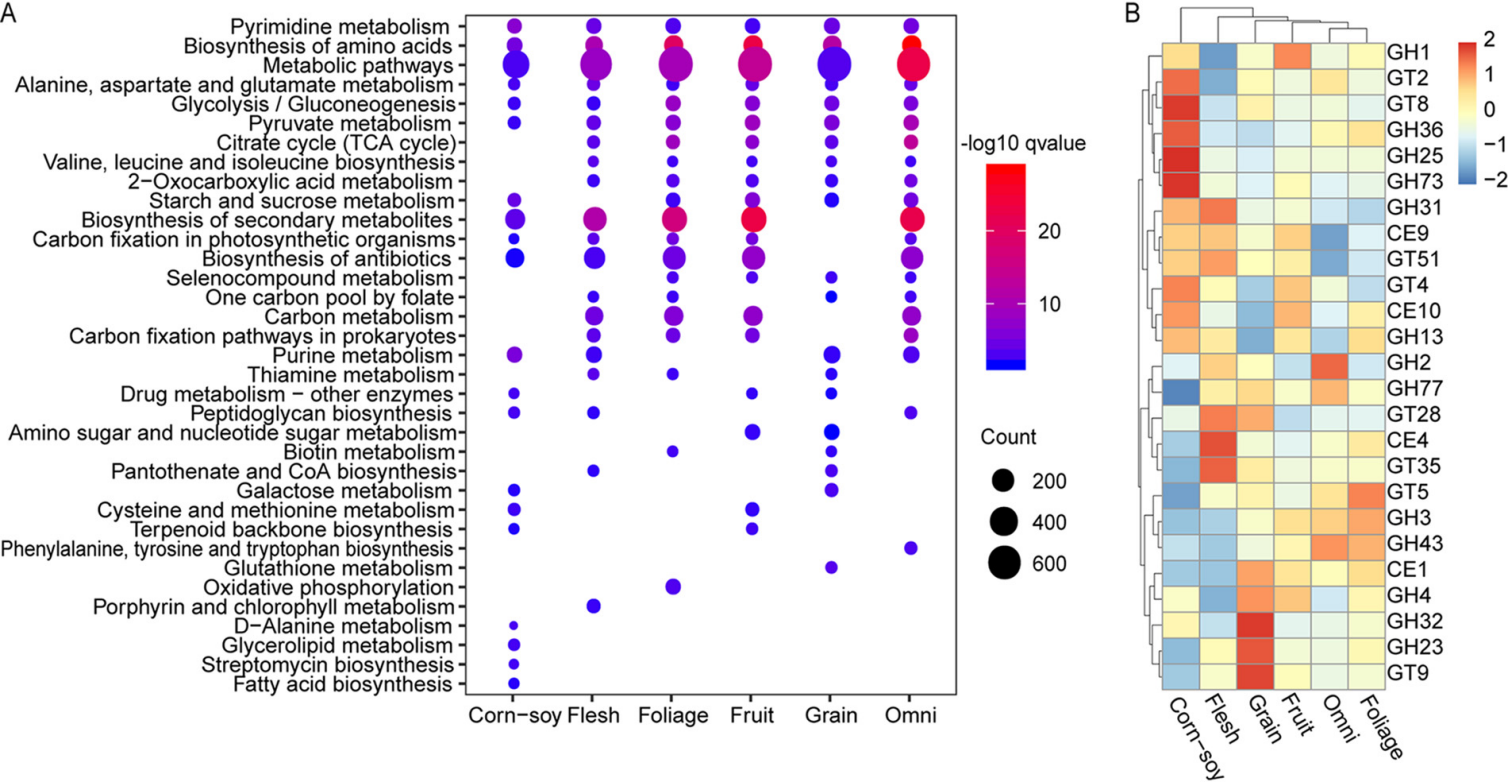
Microbial functional differences between groups. (A) KEGG pathway enrichment of the high-abundance KOs (average relative abundance > 0.01%) in each group. The top 20 most enriched pathways in each group are shown. The count equals the number of KOs in this pathway. (B) Heatmap shows the top 20 highest-abundance CAZy families in each group. The relative abundance of each CAZy family is colored according to the row z-score.

To compare the microbial functions between each group, we first tested the KEGG pathway enrichment analysis based on the top-abundance KOs (mean relative abundance > 0.01%) in each group. The top 20 significantly enriched KEGG metabolism pathways in each group are shown in Fig. 4A. Only 6 pathways were shared among the 6 groups. Due to differences in host diet, the metabolic pathway enrichment for each group was different. For example, lipid metabolism, including glycerolipid metabolism and fatty acid biosynthesis, was enriched, while some amino acid biosynthesis functions were not, in the corn-soy group. In addition, starch and sucrose metabolisms were enriched in all groups except the flesh group, a group of birds that do not eat plant-derived polysaccharides.

Moreover, we explored the distribution of CAZymes in the different groups. The top 20 abundant CAZymes in each group are shown in a z-score normalized heatmap (Fig. 4B). These data showed that most of the high-abundance CAZymes were detected in the corn-soy, flesh, and grain groups. Groups that have diets containing plant-derived fiber (fruit, “omni,” and foliage) had similar enzyme profiles and clustered together.

## DISCUSSION

Host evolutionary history and diet are suggested to be the main factors modulating microbial community composition in the vertebrate gut (10, 25). When wild animals are used to study the influence of diet and phylogeny in the composition of the gut microbiome, other factors, such as habitat, weather, and season, can bias the analysis and conclusions. In this study, we collected fecal samples from multiple species of nonpasserine birds at one time to minimize these confounding factors.

In our study, we found that diet has a greater impact on the gut microbiome than host phylogeny based on the Mantel test and a MaAsLin2 analysis (see Fig. S4 in the supplemental material). For example, different species of birds eating the same food composition tended to have similar gut microbiomes (Fig. S3). Although not all samples clustered according to food characteristics, our results support the conclusion that food source is a major factor determining the differences in intestinal microbial composition (25). The bioactivity and bioavailability of diet are two aspects driving the patterns of the nonpasserine gut microbiome assembly (28).

The predominant bacteria found in the intestinal tracts of birds fed different types of food vary greatly. The relative abundance of the *Lactobacillus*, classified in *Bacilli*, in the grain-fed group reached more than 60% of the total microbes and was significantly higher than any other group (Wilcoxon rank sum test, *P* < 0.05). Indeed, the ranking of groups, from high to low, in *Lactobacillus* abundance was grain, corn-soy, fruit, and foliage, followed by flesh and fish (Fig. 2). Starch is the major storage polysaccharide in cereal grains, legumes, and many roots and tubers (29), and the above result indicates that *Lactobacillus* was positively associated with the intake of starch-rich foods. It has been reported that starch is the only polysaccharide hydrolyzed by the extracellular enzymes (amylopullulanase) of *Lactobacillus* (30). Interestingly, functional metagenome analysis showed that genes involved in starch and sucrose metabolism were more greatly enriched in the plant-derived polysaccharide intake groups (grain, corn-soy, fruit, foliage, and “omni” food groups) than in the carnivore groups (flesh and fish groups) (Fig. 4A). Although birds can secrete pancreatic amylase, they have limited ability to digest native starch as it is highly organized (31). Taken together, we conclude that a high abundance of *Lactobacillus* in the gut microbiota of birds is essential for the metabolism of native starch.

Plant-derived fiber is the main energy source for folivores, but dietary fiber utilization by birds is inefficient and variable due to the absence of enzymes that can digest fiber (32). We found that *Clostridium* was significantly enriched in the foliage food group (Wilcoxon rank sum test, *P* < 0.05), followed by the fruit and omnivore food groups (Fig. 2C), which is consistent with the results observed in passerine birds that have plant-based diets (33). A similar CAZyme profile appeared in these 3 groups (foliage, fruit, and omnivore), whose diets contain high levels of plant cellulose (Fig. 4B). Enzymes in *Clostridium* digest fibers and produce various metabolites such as short-chain fatty acid (SCFA) that can be used by the host (34, 35); therefore, the enriched level of *Clostridium* in the guts of birds that eat plant-derived fiber could make up for the lack of fiber digestive enzymes in the host.

Foods can be regarded as a possible source of some microbes, such as the lactic acid bacteria found in the human gut microbiome (36), but it is not yet known to what extent the microbes ingested by birds with different food preferences become members of their gut microbiome. In our data, the relative abundance of *Cetobacterium*, which was identified as a gut microbe in various freshwater fish (37), was significantly higher in the fish-eating group than in the other groups (Fig. 2B). Since food eaten by piscivorous birds contains freshwater fish, the enriched level of *Cetobacterium* in these birds might be directly from fish intake. Therefore, food-derived microbes may also be one of the driving factors of the gut microbiome of some birds.

Consistent with previous studies, the phyla *Firmicutes* and *Proteobacteria* were predominant among all food groups (38–40). However, significant difference in the microbiota diversity and richness of these phyla was observed between the groups (Fig. 1D and E). In the corn-soy group, the prevalence of *Clostridia* and *Bacteroidia* resulted in a relatively high α-diversity (Fig. 1D and Fig. 3). These strong inner-connected microbes could display a complex interaction web and reveal specific microbiome diversity and function within certain environments (41, 42). Various genera, such as *Bacteroides*, *Butyricicoccus*, *Faecalibacterium*, and *Ruminiclostridium*, in the classes *Clostridia* and *Bacteroidia*, are beneficial symbionts and act as SCFA producers (43). Similarly, microbes with functions enriched for lipid metabolism, including glycerolipid metabolism and fatty acid biosynthesis, are higher in the corn-soy group than in other groups (Fig. 4A). Previous work had demonstrated that diets high in soybean proteins significantly elevate the production of SCFAs by the gut (44); thus, high soybean protein content might have led to an elevation of SCFAs in birds fed corn-soy-based diets.

In addition, the amino acid composition of natural foods is lower than in commercial feeds, which have a well-balanced amino acid profile when soybean meal and other crude protein are used as the basic ingredients (45). Moreover, essential amino acids (EAAs), which cannot be synthesized by vertebrates but can be synthesized by gut microbes (46), are usually added as additives to commercial feeds. Our data showed that gut microbiota have a strong ability to synthesize amino acids in birds that eat natural foods, with metagenomes significantly enriched for genes involved in the biosynthesis of branched-chain amino acids including valine, leucine, and isoleucine (Fig. 4A). The above result suggests that when the direct source of amino acids is limited in natural foods, then adapting the microbiome could allow enhanced synthesis of these amino acids.

Apart from digestion, gut microbes also play an important role in the health of the host. Our data showed that *Proteobacteria* was prevalent in all nonpasserine birds (Fig. 3), a result similar to previous studies in passerines (11). Most bacteria in this phylum, such as Pseudomonas, *Ralstonia*, and Acinetobacter, are Gram negative and act as pathogens or opportunistic pathogens in their hosts (47). The levels of these Gram-negative bacteria (in SC-d) were negatively related to the level of *Clostridium* (in SC-a) (Fig. 3A). Moreover, pathogens can be inhibited by SCFAs produced by *Clostridium* and *Bacteroides* (48). These characteristics might be the reason why the richness of these pathogens was significantly lower in the corn-soy group than in other groups (Fig. 3B and Table S5), further substantiating the conclusion that dietary composition influences the interactions between microbes to modulate the intestinal microbiome to reduce enteric pathogens (49).

In conclusion, we identified the role of diet in shaping the composition and function of the microbiome in nonpasserine birds. Diet type has a greater impact on gut microbiome than bird phylogeny after minimizing other complex interfering factors. Based on the analysis of microbiota diversity, cooccurrence patterns, and metabolic function, our results emphasize that the covariation of diet and gut microbiome is part of the diet-microbiome-host interaction important for the survival of birds. As we described only the association of the main types of food with the microbiome in these birds in this study, further work, including intervention studies, will be needed to fully elucidate the role of the microbiome in dietary diversity.

## MATERIALS AND METHODS

### Sample collection.

Sampling was conducted in October 2018. This study was reviewed and approved by the Animal Ethics Committee of South China Agricultural. In total, 102 fresh fecal samples were collected from 35 species of birds housed at Guangzhou Zoo, Guangzhou city, China. No antibiotic drug use was recorded for any of these birds within the 6 months prior to sampling. In addition, 27 fresh fecal samples from 6 species of domestic poultry (such as chicken, duck, and goose) were collected at nearly the same time from farms in Guangzhou. To collect fecal samples, transparent plastic film was placed on the floor of the aviary before sample collection. All fecal samples were dipped with sterile swabs from the stools of each bird immediately after defecation and then quickly placed in a 5-ml sterile sampling tubes and transported with dry ice. Samples were stored at −80°C until DNA extraction. The host phylogeny and sample metadata associated with this study are shown in Fig. 1A and in Table S1 in the supplemental material.

The birds were classified into 6 groups (omnivorous, frugivorous, granivorous, folivorous, carnivorous, and piscivorous) according to their general feeding habits. In addition, based on their food composition, the species were also divided into 7 food types: fruits (mainly apple and banana), corn-soy (commercial corn-soybean basal diet supplemented with feed additive), grains (unprocessed rice and wheat), foliage (plant stems and leaves), flesh (mainly mouse and rabbit), fish (mainly fish and loach), and omnivore (multiple types of food). In order to ensure that the bird’s nutrition is comprehensive, the breeder will regularly supplement other foods in addition to the main diet type. Detailed information on these groups is listed in Table S1.

### DNA extraction and sequencing.

For 16S rRNA gene V3-V4 hypervariable region sequencing, PCR amplicons were obtained with the primers 338F (5′-ACTCCTACGGGAGGCAGCAG-3′) and 806R (5′-GGACTACHVGGGTWTCTAAT-3′). Sequencing libraries were generated using the TruSeq Nano kit (Illumina) and assessed on the Qubit 2.0 fluorometer (Thermo Fisher Scientific) following the manufacturer’s recommendations. Sequencing was performed on an Illumina MiSeq using 2 × 250-bp paired-end V2 MiSeq reagent kits (Illumina), and an average 51,768 ± 18,927.4 tags per sample were produced.

The 16 samples selected for shotgun sequencing are listed in Table S1. Feces samples from different individuals of the same species were combined for shotgun sequencing. Bacterial cells were separated from undigested food particles and recovered through differential centrifugation before cell lysis. DNA was isolated with the Qiagen QIAamp DNA stool minikit (Qiagen, Germany) according to the manufacturer’s protocol. Metagenomic DNA paired-end libraries were prepared with an insert size of 350 bp generated with NEBNext Ultra DNA Library Prep kit for Illumina (New England Biolabs). Sequencing was performed on an Illumina Novaseq 6000 platform, with an average of 6.4 GB raw data per sample produced.

### 16S rRNA data handling.

Paired-end reads were assigned to samples based on their unique barcodes and truncated by cutting off the barcode and primer sequences using the Cutadapt (version 1.18) pipeline (50). Usearch (v11) (51) was used for removing redundant sequences and then compared with the reference Gold and SILVA databases (v132) (52) to remove chimeric and nonbacterial sequences. Sequences were clustered into operational taxonomic units (OTUs) at a similarity threshold of 97% using the UPARSE algorithm (53). OTUs were subsequently mapped to the SILVA database to determine taxonomy. General manipulation and basic analyses of the data set were performed in QIIME and R with the phyloseq, vegan, and ggplot2 packages (54, 55).

Due to the large difference in raw sequence counts, prior to running the diversity analyses, all data sets were rarefied to 5,000 total OTU counts per sample (8, 10). For within-sample microbial diversity analysis, Shannon, PD whole tree, and Chao 1 indexes were used. A nonparametric *t* test was used to calculate the significance of the α-diversity. Overall differences in the bacterial community structures were evaluated using PCoA based on the Bray-Curtis dissimilarity values, and the significance in the difference of the community compositions between groups was determined by permutational multivariate analysis of variances (PERMANOVA) using 999 permutations. UPGMA (unweighted pair group method with arithmetic mean) was performed with *upgma_cluster.py* in QIIME. The host phylogeny was obtained from http://timetree.org/. LEfSe was used to show the comparison and identify significantly different bacterial species between each group by performing a linear discriminant analysis (LDA) effect size analysis (*P* < 0.01, LDA > 4) (56).

The Mantel test was performed with the Vegan R package. Effect size and significance are derived by comparing the true data to randomly generated permutations (*n* = 9,999 for all analyses). The host phylogeny was represented by the patristic distance (branch lengths). The OTU table was converted to a Bray-Curtis dissimilarity among all pairwise sample comparisons. Euclidean distances for the diet and phylogeny data were calculated. Multivariable associations between diet, phylogeny, and OTUs were determined using MaAsLin2 (http://huttenhower.sph.harvard.edu/maaslin2) with default parameters. All tests with a Benjamini-Hochberg-adjusted *P* value of <0.05 were considered significant.

### Cooccurrence network analysis.

To reduce rare OTUs in the data set, OTUs with a relative abundance of <0.01% were removed, leaving 502 OTUs for the following analysis. Spearman’s rank coefficients (ρ) between the OTUs were calculated pairwise using the R package cooccur (57). Subsequently, significant and robust correlations (false-discovery rate [FDR] *P* value < 0.01, |ρ| ≥ 0.6) were used to construct a network using the R package igraph (58, 59). Gephi with the layout algorithm of Fruchterman-Reingold was used for network visualization (60). The relationships among the microbial taxa were estimated by establishing a correlation network, which considered both positive (Spearman’s ρ > 0.6) and negative (Spearman’s ρ < −0.6) edges. To reduce the network complexity, unclosed loops and closed loops having fewer than 4 nodes were not presented.

In total, 6 subcommunities (SC) were observed in the cooccurrence network. To assess the completeness and richness of each SC in the different individuals, the cooccurrence percentage and total abundance of an SC in each sample were calculated as follows:

$$\%=\;\frac{\text{Number of OTUs of one SC occurring}}{\text{Total number of OTUs of this SC}}\;\times \;100$$

Total abundance is the sum of the relative abundance of OTUs of one SC. The Wilcoxon rank sum test was used to statistically compare the cooccurrence percentages and abundance differences of the submicrobiota communities.

### Shotgun metagenomics data analysis.

Raw reads were cleaned to exclude adapter and low-quality sequences with fastp (v 0.19.7) (61). Contamination reads were discarded after mapping the high-quality reads to a nucleotide data set containing the chicken, maize, rice, and zebrafish genomes with BWA-MEM (v 0.7.17) (62). For each sample, clean reads were *de novo* assembled by Megahit (v1.0.3) under the pair-end mode (63). Contigs larger than 500 bp were used for open reading frame (ORF) prediction with Prodigal (v2.6.3) (64), and the predicted coding sequences (CDS) with lengths less than 102 bp were filtered out. The initial nonredundant gene set was produced by CD-HIT (65). Taxonomic assignment of the protein sequences was made on the basis of a DIAMOND (v0.8.28.90) (66) alignment against the NCBI-NR database, and genes classified as eukaryotic were excluded.

For functional profile generation, the protein sequences of all remaining genes were aligned to the Kyoto Encyclopedia of Genes and Genomes (KEGG) database and the carbohydrate-active enzyme (CAZy) database. Briefly, KEGG annotation was made on the basis of the DIAMOND alignment by taking the best hit with the criterion of an E value of <1e−5. The carbohydrate-active enzyme (CAZymes) annotation was made on the basis of an HMMER search against the dbCAN HMM (hidden Markov model) database (67).

The sequence-based gene abundance profiles were calculated by aligning the clean reads from each sample against the gene catalog through BWA-MEM with the criteria of alignment length of ≥50 bp and identity of >95% (68). Relative gene abundance profiles were summarized into KEGG and CAZy functional profiles for further analysis. KEGG pathway enrichment analysis was done with the R package clusterProfiler (69). All annotated microbial KEGG orthologies (KOs) were set as background KOs. Enriched pathways in each group were calculated based on the KOs with mean relative abundance of >0.01%. Mean abundance differences between each group in functional terms were visualized as a heatmap and clustered by hierarchical clustering using the pheatmap package in R.

### Data availability.

All the 16S rRNA amplicon sequence and shotgun metagenomic sequence generated in this study were deposited to the NCBI SRA database under the BioProject accession no. PRJNA590085.
