# Supplementary figures and images for: Covariation of the Fecal Microbiome with Diet in Nonpasserine Birds
Source: mSphere. 2021 May 12;6(3):e00308-21. doi: 10.1128/mSphere.00308-21 (PMC8125056; doi:10.1128/mSphere.00308-21)

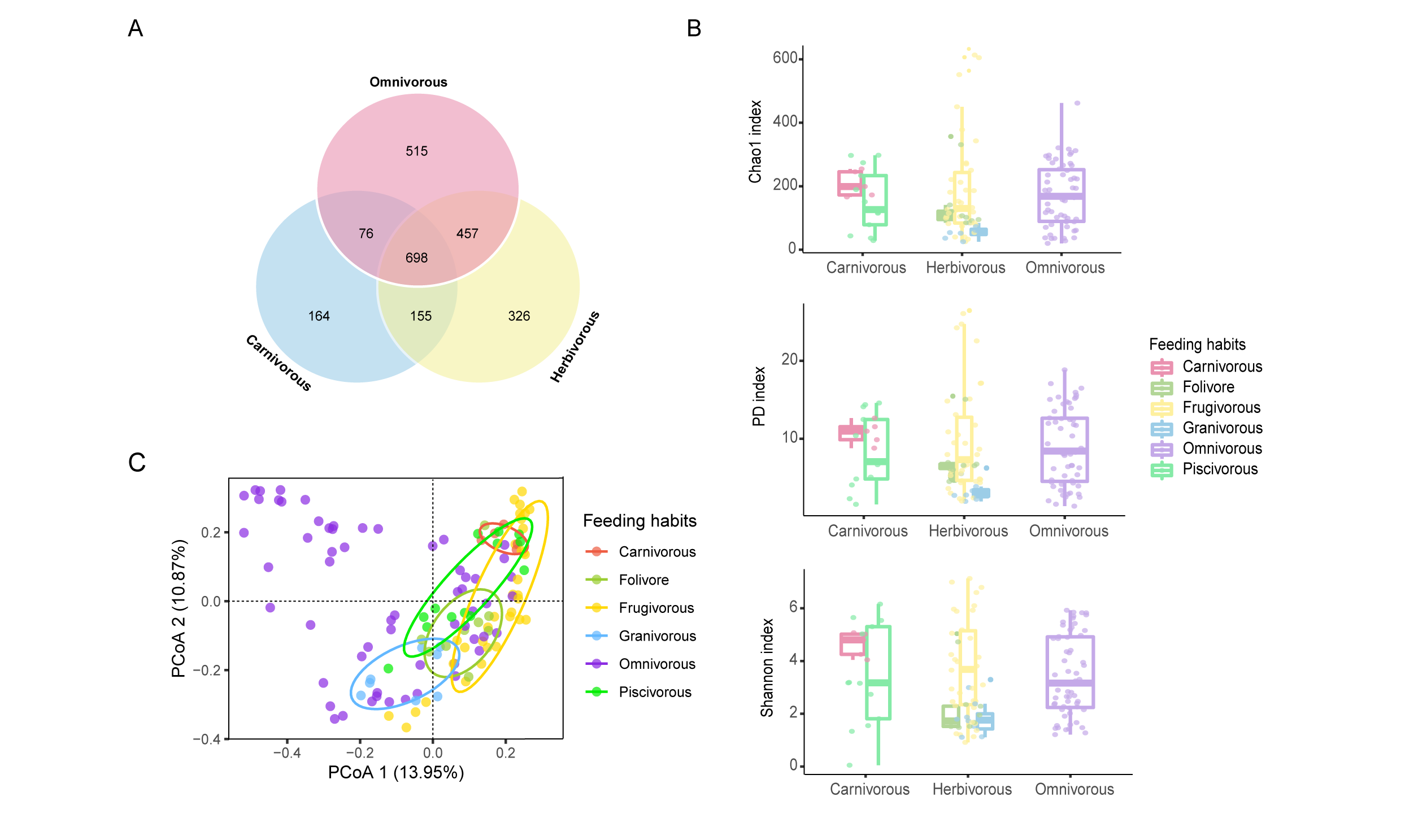

Supplement: FIG S1 [file mSphere.00308-21-sf001.tif]

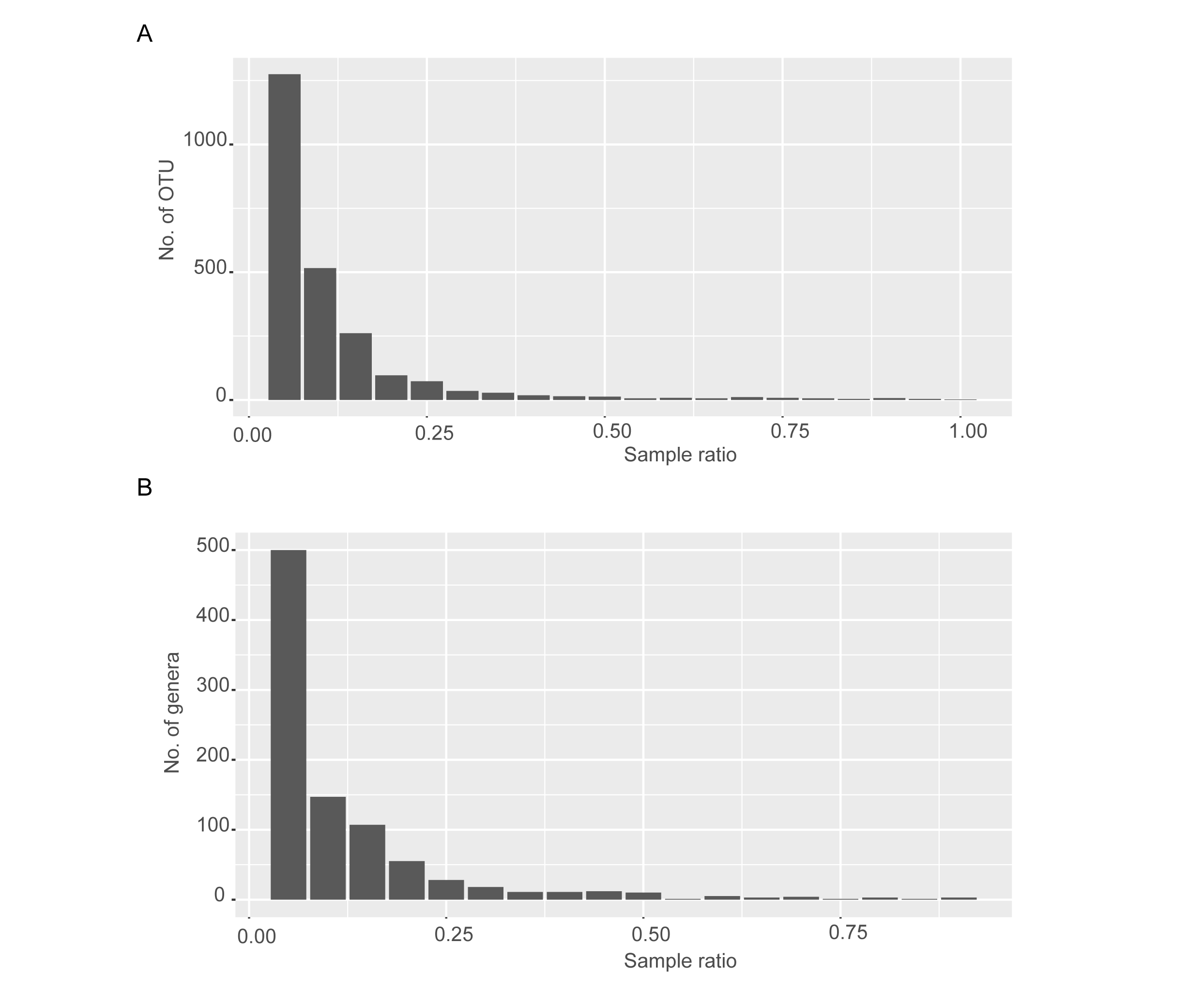

Supplement: FIG S2 [file mSphere.00308-21-sf002.tif]

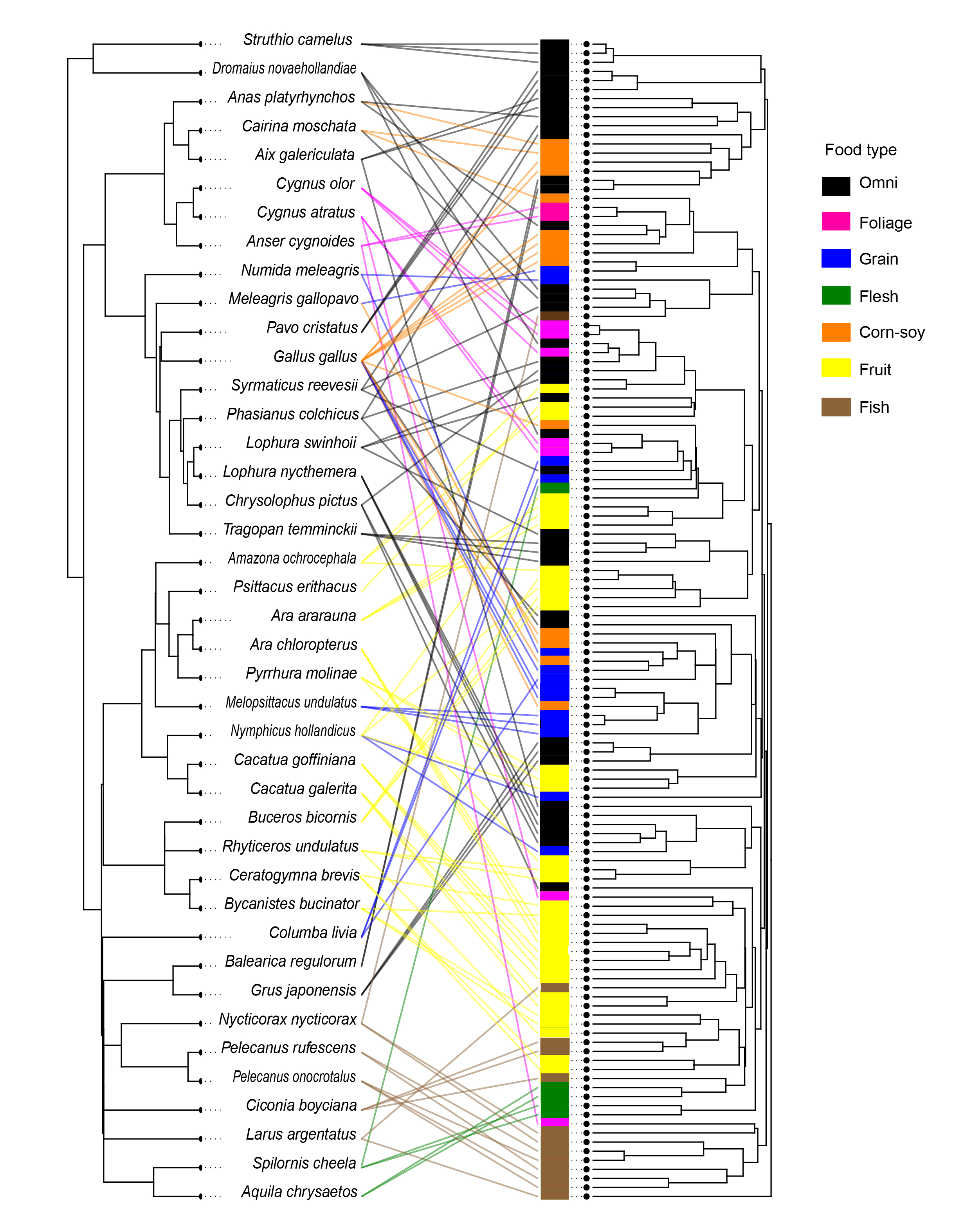

Supplement: FIG S3 [file mSphere.00308-21-sf003.jpg]

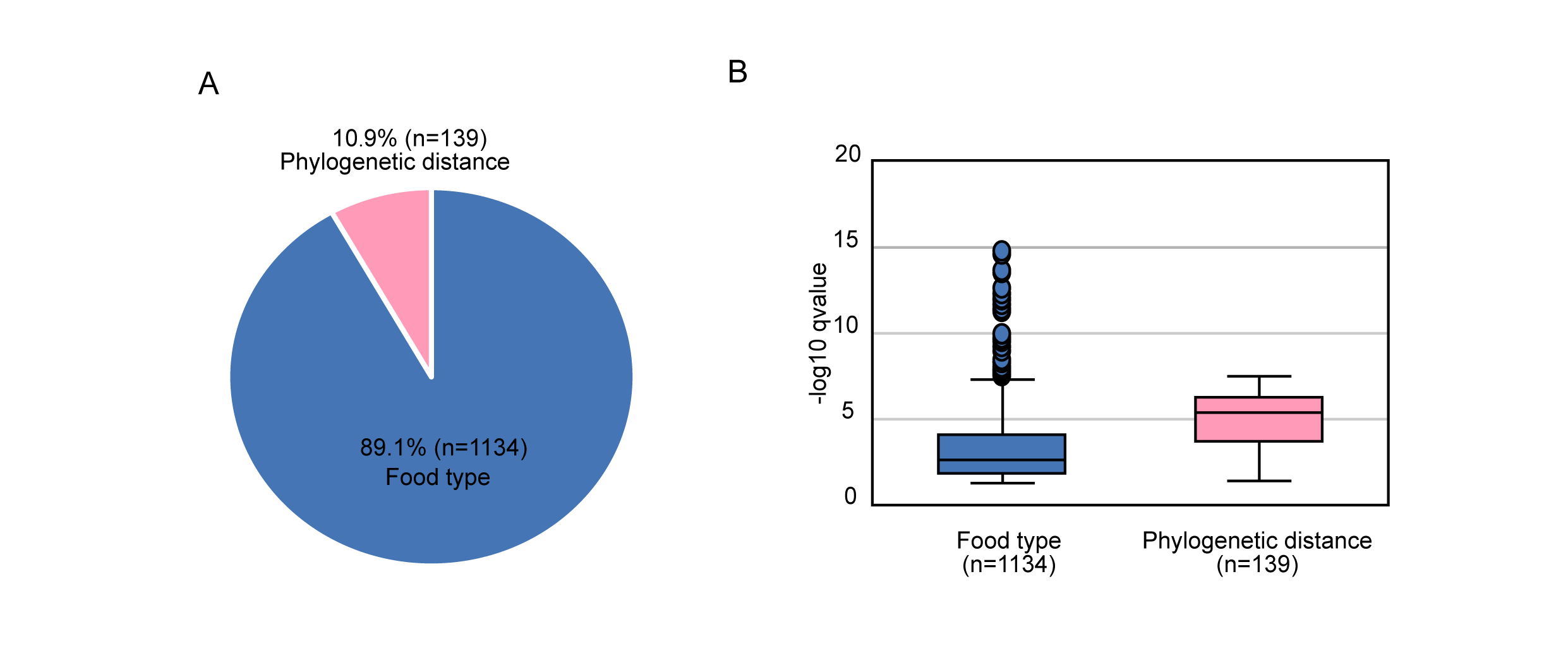

Supplement: FIG S4 [file mSphere.00308-21-sf004.tif]

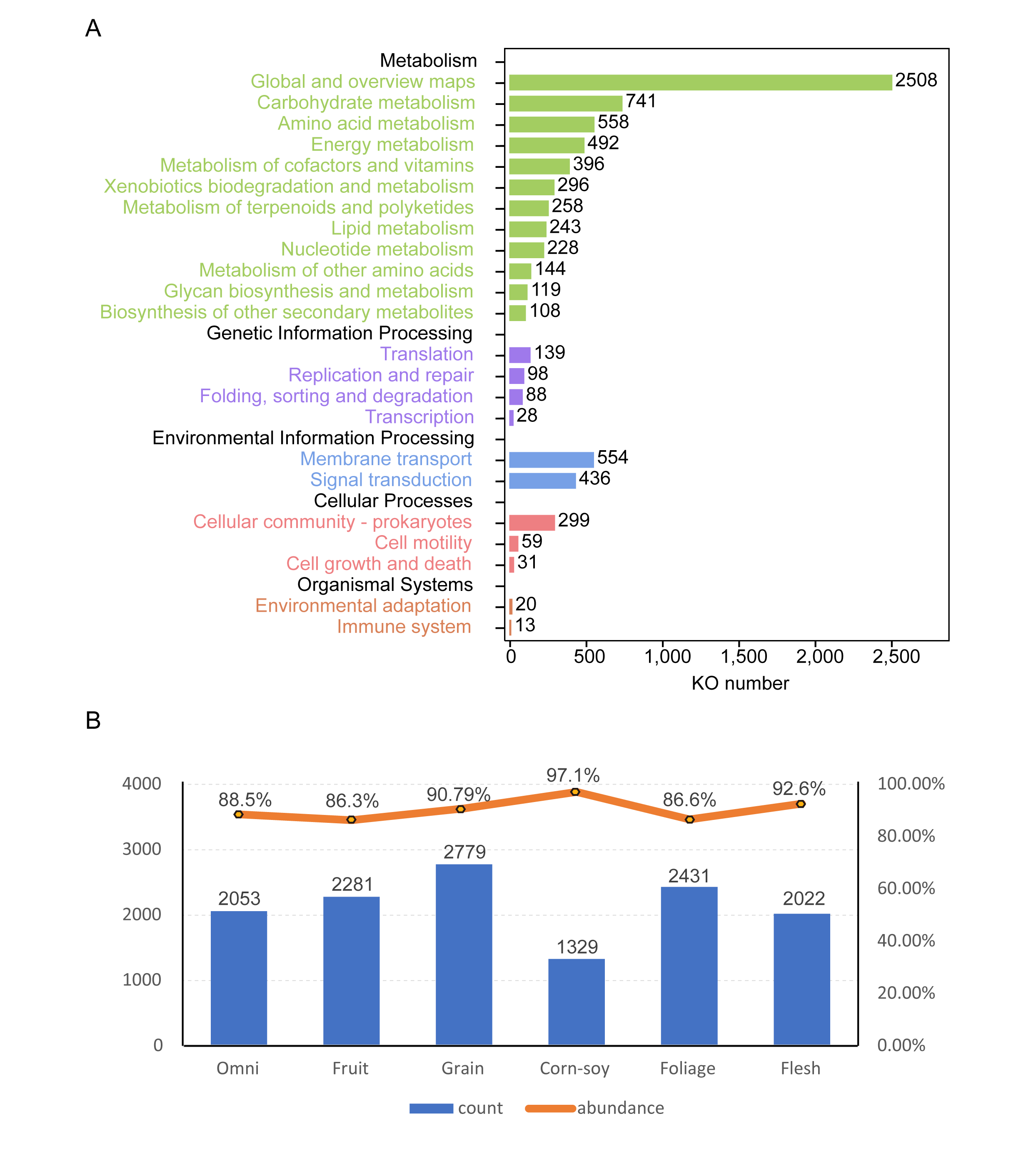

Supplement: FIG S5 [file mSphere.00308-21-sf005.tif]
