# Supplementary material for: Covariation of the Fecal Microbiome with Diet in Nonpasserine Birds
Source: mSphere. 2021 May 12;6(3):e00308-21. doi: 10.1128/mSphere.00308-21 (PMC8125056; doi:10.1128/mSphere.00308-21)
Supplement: TABLE S1 [file mSphere.00308-21-st001.docx]

**Table. S1** Metadata for each sample used in this study.

| Species | Order | Family | Sample ID  (16S) | Sample ID (shotgun) | Feeding habits | Food type | Sample position |
| --- | --- | --- | --- | --- | --- | --- | --- |
| *Balearica regulorum* | Gruiformes | Gruidae | AA1-1 AA1-2 | AvesA1 | Omnivorous | Omni | Zoo |
| *Grus japonensis* | Gruiformes | Gruidae | AA2-1 AA2-2 AA2-3 | AvesA2 | Omnivorous | Omni | Zoo |
| *Cacatua galerita* | Psittaciformes | Psittacidae | AB3-1 AB3-2 AB3-3 |  | Frugivorous | Fruit | Zoo |
| *Pyrrhura molinae* | Psittaciformes | Psittacidae | AB5-1 AB5-2 AB5-3 | AvesB5 | Frugivorous | Fruit | Zoo |
| *Cacatua goffiniana* | Psittaciformes | Psittacidae | AB6-1 AB6-2 AB6-3 |  | Frugivorous | Fruit | Zoo |
| *Psittacus erithacus* | Psittaciformes | Psittacidae | AB8-1 |  | Frugivorous | Fruit | Zoo |
| *Nymphicus hollandicus* | Psittaciformes | Psittacidae | AB9-1 AB9-2 AB9-3 |  | Frugivorous | Fruit | Zoo |
| *Ara ararauna* | Psittaciformes | Psittacidae | AB10-1 AB10-2 AB10-3 | AvesB10 | Frugivorous | Fruit | Zoo |
| *Amazona ochrocephala* | Psittaciformes | Psittacidae | AB11-1 AB11-2 AB11-3 |  | Frugivorous | Fruit | Zoo |
| *Ara chloropterus* | Psittaciformes | Psittacidae | AB12-1 AB12-2 AB12-3 | AvesB12 | Frugivorous | Fruit | Zoo |
| *Nymphicus hollandicus* | Psittaciformes | Psittacidae | AB14-1 AB14-2 |  | Granivorous | Grain | Zoo |
| *Melopsittacus undulatus* | Psittaciformes | Psittacidae | AB15-1 AB15-2 AB15-3 |  | Granivorous | Grain | Zoo |
| *Cygnus atratus* | Anseriformes | Anatidae | AC1-1 AC1-2 AC1-3 | AvesC1 | Folivore | Foliage | Zoo |
| *Aix galericulata* | Anseriformes | Anatidae | AC2-1 AC2-2 AC2-3 |  | Omnivorous | Omni | Zoo |
| *Cygnus olor* | Anseriformes | Anatidae | AC5-1 AC5-2 AC5-3 | AvesC5 | Folivore | Foliage | Zoo |
| *Cairina moschata* | Anseriformes | Anatidae | AC7-1 AC7-3 |  | Omnivorous | Corn-soy | Farm |
| *Cairina moschata* | Anseriformes | Anatidae | AC7-2 |  | Omnivorous | Omni | Farm |
| *Anas platyrhynchos* | Anseriformes | Anatidae | AC8-1 |  | Omnivorous | Omni | Farm |
| *Anas platyrhynchos* | Anseriformes | Anatidae | AC8-2 AC8-3 |  | Omnivorous | Corn-soy | Farm |
| *Anser cygnoides* | Anseriformes | Anatidae | AC9-1 AC9-2 AC9-3 |  | Folivore | Foliage | Farm |
| *Spilornis cheela* | Accipitriformes | Accipitridae | AD1-1 AD1-2 AD1-3 | AvesD1 | Carnivorous | Flesh | Zoo |
| *Aquila chrysaetos* | Accipitriformes | Accipitridae | AD2-1 AD2-2 AD2-3 |  | Carnivorous | Flesh | Zoo |
| *Lophura swinhoii* | Galliformes | Phasianidae | AE1-1 AE1-2 AE1-3 |  | Omnivorous | Omni | Zoo |
| *Lophura nycthemera* | Galliformes | Phasianidae | AE2-1 AE2-2 AE2-3 |  | Omnivorous | Omni | Zoo |
| *Chrysolophus pictus* | Galliformes | Phasianidae | AE3-1 AE3-2 AE3-3 |  | Omnivorous | Omni | Zoo |
| *Pavo cristatus* | Galliformes | Phasianidae | AE4-1 AE4-2 AE4-3 | AvesE4 | Omnivorous | Omni | Zoo |
| *Tragopan temminckii* | Galliformes | Phasianidae | AE5-1 AE5-2 AE5-3 |  | Omnivorous | Omni | Zoo |
| *Syrmaticus reevesii* | Galliformes | Phasianidae | AE6-1 AE6-2 AE6-3 |  | Omnivorous | Omni | Zoo |
| *Phasianus colchicus* | Galliformes | Phasianidae | AE7-1 AE7-2 AE7-3 | AvesE7 | Omnivorous | Omni | Zoo |
| *Gallus gallus domesticus* | Galliformes | Phasianidae | AE8-1 AE8-2 AE8-3 AE9-1 AE9-2 AE9-3 AE10-1 AE10-2 AE10-3 AE11-2 | AvesE10 | Omnivorous | Corn-soy | Farm |
| *Gallus gallus domesticus* | Galliformes | Phasianidae | AE11-1 |  | Omnivorous | Omni | Farm |
| *Gallus gallus domesticus* | Galliformes | Phasianidae | AE11-3 AE11-4 AE14-1 |  | Omnivorous | Grain | Farm |
| *Meleagris gallopavo* | Galliformes | Phasianidae | AE12-1 | AvesE12 | Omnivorous | Grain | Farm |
| *Meleagris gallopavo* | Galliformes | Phasianidae | AE12-2 |  | Omnivorous | Corn-soy | Farm |
| *Numida meleagris* | Galliformes | Numididae | AE13-1 AE13-2 |  | Omnivorous | Grain | Farm |
| *Nycticorax nycticorax* | Pelecaniformes | Ardeidae | AG6-1 AG6-2 AG6-3 |  | Piscivorous | Fish | Zoo |
| *Ciconia boyciana* | Ciconiiformes | Ciconiidae | AG7-1 AG7-2 AG7-3 |  | Piscivorous | Fish | Zoo |
| *Ceratogymna bucinator* | Bucerotiformes | Bucerotidae | AJ1-1  AJ1-2  AJ1-3 |  | Frugivorous | Fruit | Zoo |
| *Rhyticeros undulatus* | Bucerotiformes | Bucerotidae | AJ2-1  AJ2-2  AJ2-3 |  | Frugivorous | Fruit | Zoo |
| *Buceros bicornis* | Bucerotiformes | Bucerotidae | AJ3-1  AJ3-2  AJ3-3 |  | Frugivorous | Fruit | Zoo |
| *Ceratogymna brevis* | Bucerotiformes | Bucerotidae | AJ4-1  AJ4-2  AJ4-3 | AvesJ4 | Frugivorous | Fruit | Zoo |
| *Struthio camelus* | Struthioniformes | Struthionidae | AM1-1 AM1-2 AM1-3 | AvesM1 | Omnivorous | Omni | Zoo |
| *Dromaius novaehollandiae* | Casuariiformes | Dromaiidae | AN1-1 AN1-2 AN1-3 | AvesN1 | Omnivorous | Omni | Zoo |
| *Columba livia* | Columbiformes | Columbidae | AQ1-1 AQ1-2 AQ1-3 | AvesQ1 | Granivorous | Grain | Zoo |
| *Larus argentatus* | Charadriiformes | Laridae | AK1-1 AK1-2 |  | Piscivorous | Fish | Zoo |
| *Pelecanus onocrotalus* | Pelecaniformes | Pelecanidae | AL1-1 AL1-2 AL1-3 |  | Piscivorous | Fish | Zoo |
| *Pelecanus rufescens* | Pelecaniformes | Pelecanidae | AL2-1 AL2-2 |  | Piscivorous | Fish | Zoo |
