# Supplementary material for: Covariation of the Fecal Microbiome with Diet in Nonpasserine Birds
Source: mSphere. 2021 May 12;6(3):e00308-21. doi: 10.1128/mSphere.00308-21 (PMC8125056; doi:10.1128/mSphere.00308-21)
Supplement: TABLE S2 [file mSphere.00308-21-st002.docx]

**Table. S2** Comparison of the α-diversity (Chao1 index, PD whole tree index and Shannon index) between the pairwise groups.

| α-diversity | Chao 1 index | | | | PD whole tree index | | | | Shannon index | | | |
| --- | --- | --- | --- | --- | --- | --- | --- | --- | --- | --- | --- | --- |
|  | diff | lwr | upr | p adj | diff | lwr | upr | p adj | diff | lwr | upr | p adj |
| Fish-Corn-soy | -61.13667 | -187.4334 | 65.160089 | 0.7724946 | -2.514591 | -8.181575 | 3.1523925 | 0.836286 | -0.427746 | -2.19741 | 1.341923 | 0.9908182 |
| Flesh-Corn-soy | -6.581786 | -178.6953 | 165.53168 | 0.9999998 | 0.199207 | -7.52359 | 7.9220044 | 1 | 0.7729087 | -1.63874 | 3.18456 | 0.9612618 |
| Flesh-Fish | 54.55488 | -120.8372 | 229.947 | 0.9665198 | 2.713799 | -5.156113 | 10.58371 | 0.945094 | 1.2006543 | -1.25694 | 3.658246 | 0.7648296 |
| Foliage-Corn-soy | -82.08019 | -222.6102 | 58.449871 | 0.5831837 | -3.300928 | -9.606565 | 3.0047096 | 0.701734 | -1.727805 | -3.69691 | 0.2413 | 0.1258928 |
| Foliage-Fish | -20.94352 | -165.4705 | 123.58345 | 0.9994685 | -0.786336 | -7.271317 | 5.6986439 | 0.999811 | -1.30006 | -3.32517 | 0.72505 | 0.4680369 |
| Foliage-Flesh | -75.4984 | -261.4022 | 110.40539 | 0.8860226 | -3.500135 | -11.84171 | 4.8414391 | 0.869346 | -2.500714 | -5.1056 | 0.104167 | 0.0686993 |
| Fruit-Corn-soy | -21.65783 | -124.9681 | 81.652429 | 0.9957469 | -0.919407 | -5.554978 | 3.7161638 | 0.996866 | -0.129405 | -1.57699 | 1.318177 | 0.9999683 |
| Fruit-Fish | 39.47884 | -69.20583 | 148.16351 | 0.9301972 | 1.595184 | -3.281538 | 6.4719069 | 0.957251 | 0.2983409 | -1.22455 | 1.821229 | 0.9970733 |
| Fruit-Flesh | -15.07604 | -174.7148 | 144.56271 | 0.9999562 | -1.118614 | -8.281666 | 6.0444377 | 0.999184 | -0.902313 | -3.13917 | 1.334543 | 0.8892383 |
| Fruit-Foliage | 60.42236 | -64.51836 | 185.36308 | 0.7732817 | 2.381521 | -3.224617 | 7.9876587 | 0.862559 | 1.5984007 | -0.15227 | 3.349068 | 0.0978083 |
| Grain-Corn-soy * | -154.2443 | -278.1011 | -30.38749 | 0.0052297 | -7.311523 | -12.86902 | -1.754021 | 0.002511 | -2.010534 | -3.74601 | -0.275054 | 0.0123096 |
| Grain-Fish | -93.10761 | -221.4815 | 35.266271 | 0.3169412 | -4.796932 | -10.55712 | 0.9632534 | 0.169611 | -1.582788 | -3.38156 | 0.215985 | 0.1237584 |
| Grain-Flesh * | -147.6625 | -321.3059 | 25.980901 | 0.1512079 | -7.51073 | -15.30218 | 0.2807151 | 0.066617 | -2.783443 | -5.21653 | -0.350354 | 0.0141179 |
| Grain-Foliage | -72.16409 | -214.5638 | 70.235628 | 0.7325972 | -4.010595 | -10.40013 | 2.3789345 | 0.496086 | -0.282729 | -2.27803 | 1.712574 | 0.9995328 |
| Grain-Fruit * | -132.5865 | -238.4259 | -26.74699 | 0.0048506 | -6.392116 | -11.14117 | -1.643059 | 0.001809 | -1.881129 | -3.36415 | -0.398108 | 0.0041251 |
| Omni-Corn-soy | -42.89261 | -144.5247 | 58.739515 | 0.8661928 | -1.443749 | -6.004021 | 3.1165232 | 0.963497 | -0.354422 | -1.77849 | 1.069646 | 0.9892788 |
| Omni-Fish | 18.24406 | -88.84673 | 125.33485 | 0.9986623 | 1.070842 | -3.734362 | 5.8760467 | 0.994065 | 0.0733233 | -1.42723 | 1.573878 | 0.9999991 |
| Omni-Flesh | -36.31082 | -194.8687 | 122.24709 | 0.9931234 | -1.642956 | -8.75751 | 5.4715977 | 0.992807 | -1.127331 | -3.34904 | 1.09438 | 0.7314292 |
| Omni-Foliage | 39.18758 | -84.36914 | 162.7443 | 0.9631759 | 1.857179 | -3.686858 | 7.4012159 | 0.952102 | 1.3733831 | -0.35789 | 3.104658 | 0.2167342 |
| Omni-Fruit | -21.23478 | -99.91499 | 57.445426 | 0.9836286 | -0.524342 | -4.054753 | 3.0060688 | 0.999388 | -0.225018 | -1.32748 | 0.877448 | 0.9963286 |
| Omni-Grain * | 111.3517 | 7.1496029 | 215.55375 | 0.0279987 | 5.867774 | 1.1921874 | 10.543361 | 0.004739 | 1.6561117 | 0.196034 | 3.11619 | 0.0154643 |
