# Supplementary material for: Covariation of the Fecal Microbiome with Diet in Nonpasserine Birds
Source: mSphere. 2021 May 12;6(3):e00308-21. doi: 10.1128/mSphere.00308-21 (PMC8125056; doi:10.1128/mSphere.00308-21)
Supplement: TABLE S3 [file mSphere.00308-21-st003.docx]

**Table. S3** List of a set of topological metrics of the co-occurrence network.

| No. of nodes | No. of edge | Average degree | Average weighted degree | Average path length | Density | Average Clustering Coefficient |
| --- | --- | --- | --- | --- | --- | --- |
| 285 | 2689 | 18.870 | 12.900 | 3.401 | 0.066 | 0.660 |
