# Supplementary material for: Covariation of the Fecal Microbiome with Diet in Nonpasserine Birds
Source: mSphere. 2021 May 12;6(3):e00308-21. doi: 10.1128/mSphere.00308-21 (PMC8125056; doi:10.1128/mSphere.00308-21)
Supplement: TABLE S4 [file mSphere.00308-21-st004.docx]

**Table. S4** Taxonomic annotation of the OTUs in each sub-network.

|  | phylum | class | order | family | genus |
| --- | --- | --- | --- | --- | --- |
| **SC-a** |  |  |  |  |  |
| OTU_93 | D_1_Bacteroidetes | D_2_Bacteroidia | D_3_Bacteroidales | D_4_Bacteroidaceae | D_5_Bacteroides |
| OTU_84 | D_1_Proteobacteria | D_2_Gammaproteobacteria | D_3_Aeromonadales | D_4_Succinivibrionaceae | D_5_Anaerobiospirillum |
| OTU_35 | D_1_Bacteroidetes | D_2_Bacteroidia | D_3_Bacteroidales | D_4_Bacteroidaceae | D_5_Bacteroides |
| OTU_492 | D_1_Bacteroidetes | D_2_Bacteroidia | D_3_Bacteroidales | D_4_Prevotellaceae | D_5_Prevotellaceae UCG-001 |
| OTU_518 | D_1_Bacteroidetes | D_2_Bacteroidia | D_3_Bacteroidales | D_4_Prevotellaceae | D_5_Prevotellaceae Ga6A1 group |
| OTU_127 | D_1_Firmicutes | D_2_Clostridia | D_3_Clostridiales | D_4_Ruminococcaceae | D_5_Faecalibacterium |
| OTU_120 | D_1_Bacteroidetes | D_2_Bacteroidia | D_3_Bacteroidales | D_4_Rikenellaceae | D_5_Rikenellaceae RC9 gut group |
| OTU_1934 | D_1_Firmicutes | D_2_Clostridia | D_3_Clostridiales | D_4_Lachnospiraceae | D_5_CAG-56 |
| OTU_475 | D_1_Bacteroidetes | D_2_Bacteroidia | D_3_Bacteroidales |  |  |
| OTU_620 | D_1_Bacteroidetes | D_2_Bacteroidia | D_3_Bacteroidales | D_4_Prevotellaceae | D_5_Prevotellaceae UCG-001 |
| OTU_76 | D_1_Bacteroidetes | D_2_Bacteroidia | D_3_Bacteroidales | D_4_Bacteroidaceae | D_5_Bacteroides |
| OTU_114 | D_1_Bacteroidetes | D_2_Bacteroidia | D_3_Bacteroidales | D_4_Rikenellaceae | D_5_Rikenellaceae RC9 gut group |
| OTU_318 | D_1_Proteobacteria | D_2_Deltaproteobacteria | D_3_Desulfovibrionales | D_4_Desulfovibrionaceae | D_5_Desulfovibrio |
| OTU_442 | D_1_Firmicutes | D_2_Clostridia | D_3_Clostridiales | D_4_Ruminococcaceae | D_5_Ruminiclostridium 5 |
| OTU_311 | D_1_Bacteroidetes | D_2_Bacteroidia | D_3_Bacteroidales | D_4_Bacteroidaceae | D_5_Bacteroides |
| OTU_106 | D_1_Bacteroidetes | D_2_Bacteroidia | D_3_Bacteroidales | D_4_Bacteroidaceae | D_5_Bacteroides |
| OTU_117 | D_1_Actinobacteria | D_2_Coriobacteriia | D_3_Coriobacteriales | D_4_Coriobacteriaceae | D_5_Collinsella |
| OTU_303 | D_1_Firmicutes | D_2_Clostridia | D_3_Clostridiales | D_4_Ruminococcaceae | D_5_Oscillospira |
| OTU_277 | D_1_Firmicutes | D_2_Clostridia | D_3_Clostridiales | D_4_Ruminococcaceae | D_5_Subdoligranulum |
| OTU_1742 | D_1_Firmicutes | D_2_Clostridia | D_3_Clostridiales | D_4_Ruminococcaceae | D_5_Ruminococcaceae UCG-014 |
| OTU_580 | D_1_Bacteroidetes | D_2_Bacteroidia | D_3_Bacteroidales | D_4_Muribaculaceae | D_5_uncultured bacterium |
| OTU_237 | D_1_Bacteroidetes | D_2_Bacteroidia | D_3_Bacteroidales | D_4_Rikenellaceae |  |
| OTU_402 | D_1_Firmicutes | D_2_Clostridia | D_3_Clostridiales | D_4_Ruminococcaceae | D_5_[Eubacterium] coprostanoligenes group |
| OTU_134 | D_1_Bacteroidetes | D_2_Bacteroidia | D_3_Bacteroidales | D_4_Rikenellaceae | D_5_Rikenellaceae RC9 gut group |
| OTU_435 | D_1_Bacteroidetes | D_2_Bacteroidia | D_3_Bacteroidales | D_4_Prevotellaceae | D_5_Prevotellaceae UCG-001 |
| OTU_495 | D_1_Firmicutes | D_2_Clostridia | D_3_Clostridiales | D_4_Ruminococcaceae | D_5_uncultured |
| OTU_877 | D_1_Bacteroidetes | D_2_Bacteroidia | D_3_Bacteroidales | D_4_Bacteroidaceae | D_5_Bacteroides |
| OTU_1163 | D_1_Firmicutes | D_2_Clostridia | D_3_Clostridiales | D_4_Ruminococcaceae | D_5_uncultured |
| OTU_460 | D_1_Bacteroidetes | D_2_Bacteroidia | D_3_Bacteroidales | D_4_Prevotellaceae | D_5_Prevotellaceae UCG-001 |
| OTU_834 | D_1_Bacteroidetes | D_2_Bacteroidia | D_3_Bacteroidales | D_4_Muribaculaceae | D_5_uncultured bacterium |
| OTU_214 | D_1_Bacteroidetes | D_2_Bacteroidia | D_3_Bacteroidales | D_4_Bacteroidaceae | D_5_Bacteroides |
| OTU_2937 | D_1_Firmicutes | D_2_Clostridia | D_3_Clostridiales | D_4_Lachnospiraceae | D_5_Lachnoclostridium |
| OTU_375 | D_1_Bacteroidetes | D_2_Bacteroidia | D_3_Bacteroidales | D_4_Bacteroidaceae | D_5_Bacteroides |
| OTU_404 | D_1_Bacteroidetes | D_2_Bacteroidia | D_3_Bacteroidales | D_4_Rikenellaceae | D_5_Rikenellaceae RC9 gut group |
| OTU_1441 | D_1_Bacteroidetes | D_2_Bacteroidia | D_3_Bacteroidales | D_4_Tannerellaceae | D_5_Parabacteroides |
| OTU_81 | D_1_Actinobacteria | D_2_Coriobacteriia | D_3_Coriobacteriales | D_4_Atopobiaceae | D_5_Olsenella |
| OTU_356 | D_1_Bacteroidetes | D_2_Bacteroidia | D_3_Bacteroidales | D_4_Prevotellaceae | D_5_uncultured |
| OTU_135 | D_1_Firmicutes | D_2_Clostridia | D_3_Clostridiales | D_4_Ruminococcaceae | D_5_Subdoligranulum |
| OTU_1107 | D_1_Firmicutes | D_2_Clostridia | D_3_Clostridiales | D_4_Ruminococcaceae | D_5_Anaerofilum |
| OTU_744 | D_1_Bacteroidetes | D_2_Bacteroidia | D_3_Bacteroidales | D_4_Bacteroidaceae | D_5_Bacteroides |
| OTU_662 | D_1_Bacteroidetes | D_2_Bacteroidia | D_3_Bacteroidales | D_4_Bacteroidaceae | D_5_Bacteroides |
| OTU_207 | D_1_Firmicutes | D_2_Clostridia | D_3_Clostridiales | D_4_Lachnospiraceae | D_5_Shuttleworthia |
| OTU_904 | D_1_Bacteroidetes | D_2_Bacteroidia | D_3_Bacteroidales | D_4_Prevotellaceae | D_5_Alloprevotella |
| OTU_1408 | D_1_Firmicutes | D_2_Clostridia | D_3_Clostridiales | D_4_Ruminococcaceae | D_5_Ruminococcaceae UCG-014 |
| OTU_499 | D_1_Proteobacteria | D_2_Gammaproteobacteria | D_3_Betaproteobacteriales | D_4_Burkholderiaceae | D_5_Parasutterella |
| OTU_837 | D_1_Bacteroidetes | D_2_Bacteroidia | D_3_Bacteroidales | D_4_Rikenellaceae | D_5_Rikenellaceae RC9 gut group |
| OTU_111 | D_1_Firmicutes | D_2_Clostridia | D_3_Clostridiales | D_4_Ruminococcaceae | D_5_Fournierella |
| OTU_46 | D_1_Proteobacteria | D_2_Deltaproteobacteria | D_3_Desulfovibrionales | D_4_Desulfovibrionaceae | D_5_Desulfovibrio |
| OTU_545 | D_1_Bacteroidetes | D_2_Bacteroidia | D_3_Bacteroidales | D_4_Rikenellaceae | D_5_Alistipes |
| OTU_401 | D_1_Bacteroidetes | D_2_Bacteroidia | D_3_Bacteroidales | D_4_Tannerellaceae | D_5_Parabacteroides |
| OTU_235 | D_1_Patescibacteria | D_2_Saccharimonadia | D_3_Saccharimonadales |  |  |
| OTU_952 | D_1_Bacteroidetes | D_2_Bacteroidia | D_3_Bacteroidales | D_4_Prevotellaceae | D_5_Prevotellaceae UCG-001 |
| OTU_40 | D_1_Firmicutes | D_2_Clostridia | D_3_Clostridiales | D_4_Ruminococcaceae | D_5_Ruminococcaceae UCG-005 |
| OTU_965 | D_1_Bacteroidetes | D_2_Bacteroidia | D_3_Bacteroidales |  |  |
| OTU_52 | D_1_Firmicutes | D_2_Clostridia | D_3_Clostridiales | D_4_Ruminococcaceae | D_5_Subdoligranulum |
| OTU_493 | D_1_Bacteroidetes | D_2_Bacteroidia | D_3_Bacteroidales | D_4_Bacteroidaceae | D_5_Bacteroides |
| OTU_1377 | D_1_Firmicutes | D_2_Negativicutes | D_3_Selenomonadales | D_4_Acidaminococcaceae | D_5_Phascolarctobacterium |
| OTU_222 | D_1_Firmicutes | D_2_Clostridia | D_3_Clostridiales | D_4_Ruminococcaceae | D_5_Ruminococcaceae UCG-014 |
| OTU_600 | D_1_Firmicutes | D_2_Clostridia | D_3_Clostridiales | D_4_Ruminococcaceae | D_5_Negativibacillus |
| OTU_227 | D_1_Firmicutes | D_2_Erysipelotrichia | D_3_Erysipelotrichales | D_4_Erysipelotrichaceae | D_5_Faecalitalea |
| OTU_825 | D_1_Proteobacteria | D_2_Gammaproteobacteria | D_3_Aeromonadales | D_4_Succinivibrionaceae | D_5_Succinatimonas |
| OTU_1068 | D_1_Bacteroidetes | D_2_Bacteroidia | D_3_Bacteroidales | D_4_Muribaculaceae | D_5_uncultured bacterium |
| OTU_1218 | D_1_Firmicutes | D_2_Clostridia | D_3_Clostridiales | D_4_Lachnospiraceae | D_5_Shuttleworthia |
| OTU_959 | D_1_Firmicutes | D_2_Clostridia | D_3_Clostridiales | D_4_Ruminococcaceae |  |
| OTU_619 | D_1_Firmicutes | D_2_Clostridia | D_3_Clostridiales | D_4_Lachnospiraceae | D_5_Oribacterium |
| OTU_183 | D_1_Firmicutes | D_2_Negativicutes | D_3_Selenomonadales | D_4_Veillonellaceae | D_5_Megamonas |
| OTU_335 | D_1_Firmicutes | D_2_Erysipelotrichia | D_3_Erysipelotrichales | D_4_Erysipelotrichaceae | D_5_Faecalitalea |
| OTU_671 | D_1_Bacteroidetes | D_2_Bacteroidia | D_3_Bacteroidales | D_4_Barnesiellaceae |  |
| OTU_546 | D_1_Firmicutes | D_2_Clostridia | D_3_Clostridiales | D_4_Lachnospiraceae | D_5_Lachnospiraceae FCS020 group |
| OTU_878 | D_1_Bacteroidetes | D_2_Bacteroidia | D_3_Bacteroidales |  |  |
| OTU_1120 | D_1_Bacteroidetes | D_2_Bacteroidia | D_3_Bacteroidales | D_4_Bacteroidaceae | D_5_Bacteroides |
| OTU_3406 | D_1_Firmicutes | D_2_Clostridia | D_3_Clostridiales | D_4_Ruminococcaceae | D_5_Ruminococcaceae UCG-014 |
| OTU_213 | D_1_Firmicutes | D_2_Clostridia | D_3_Clostridiales | D_4_Ruminococcaceae |  |
| OTU_3266 | D_1_Proteobacteria | D_2_Deltaproteobacteria | D_3_Desulfovibrionales | D_4_Desulfovibrionaceae | D_5_Desulfovibrio |
| OTU_750 | D_1_Bacteroidetes | D_2_Bacteroidia | D_3_Bacteroidales | D_4_Prevotellaceae |  |
| OTU_699 | D_1_Bacteroidetes | D_2_Bacteroidia | D_3_Bacteroidales | D_4_Prevotellaceae | D_5_Prevotellaceae UCG-001 |
| OTU_595 | D_1_Firmicutes | D_2_Clostridia | D_3_Clostridiales | D_4_Christensenellaceae | D_5_Christensenellaceae R-7 group |
| OTU_512 | D_1_Verrucomicrobia | D_2_Verrucomicrobiae | D_3_Verrucomicrobiales | D_4_Akkermansiaceae | D_5_Akkermansia |
| OTU_940 | D_1_Proteobacteria | D_2_Deltaproteobacteria | D_3_Desulfovibrionales | D_4_Desulfovibrionaceae | D_5_Desulfovibrio |
| OTU_774 | D_1_Firmicutes | D_2_Clostridia | D_3_Clostridiales | D_4_Lachnospiraceae | D_5_uncultured |
| OTU_836 | D_1_Firmicutes | D_2_Clostridia | D_3_Clostridiales | D_4_Ruminococcaceae |  |
| OTU_1045 | D_1_Bacteroidetes | D_2_Bacteroidia | D_3_Bacteroidales | D_4_Tannerellaceae | D_5_Parabacteroides |
| OTU_574 | D_1_Bacteroidetes | D_2_Bacteroidia | D_3_Bacteroidales | D_4_Tannerellaceae | D_5_Parabacteroides |
| OTU_811 | D_1_Bacteroidetes | D_2_Bacteroidia | D_3_Bacteroidales | D_4_Marinifilaceae | D_5_Odoribacter |
| OTU_591 | D_1_Bacteroidetes | D_2_Bacteroidia | D_3_Bacteroidales | D_4_Barnesiellaceae | D_5_Barnesiella |
| OTU_551 | D_1_Bacteroidetes | D_2_Bacteroidia | D_3_Bacteroidales | D_4_uncultured | Ambiguous_taxa |
| OTU_302 | D_1_Firmicutes | D_2_Clostridia | D_3_Clostridiales | D_4_Christensenellaceae | D_5_Christensenellaceae R-7 group |
| OTU_521 | D_1_Firmicutes | D_2_Clostridia | D_3_Clostridiales | D_4_Lachnospiraceae | D_5_Marvinbryantia |
| OTU_1174 | D_1_Bacteroidetes | D_2_Bacteroidia | D_3_Bacteroidales | D_4_Tannerellaceae | D_5_Parabacteroides |
| OTU_324 | D_1_Bacteroidetes | D_2_Bacteroidia | D_3_Bacteroidales | D_4_Bacteroidaceae | D_5_Bacteroides |
| OTU_3554 | D_1_Elusimicrobia | D_2_Elusimicrobia | D_3_Elusimicrobiales | D_4_Elusimicrobiaceae | D_5_Elusimicrobium |
| OTU_2597 | D_1_Firmicutes | D_2_Clostridia | D_3_Clostridiales | D_4_Lachnospiraceae | D_5_Lachnospiraceae NK4A136 group |
| OTU_920 | D_1_Actinobacteria | D_2_Coriobacteriia | D_3_Coriobacteriales | D_4_Coriobacteriaceae | D_5_Enorma |
| OTU_740 | D_1_Firmicutes | D_2_Clostridia | D_3_Clostridiales | D_4_Ruminococcaceae | D_5_Ruminococcaceae UCG-014 |
| OTU_456 | D_1_Spirochaetes | D_2_Spirochaetia | D_3_Spirochaetales | D_4_Spirochaetaceae |  |
| OTU_279 | D_1_Actinobacteria | D_2_Coriobacteriia | D_3_Coriobacteriales | D_4_Eggerthellaceae | D_5_Slackia |
| OTU_173 | D_1_Firmicutes | D_2_Clostridia | D_3_Clostridiales | D_4_Lachnospiraceae | D_5_CHKCI001 |
| OTU_389 | D_1_Firmicutes | D_2_Erysipelotrichia | D_3_Erysipelotrichales | D_4_Erysipelotrichaceae | D_5_Erysipelatoclostridium |
| OTU_553 | D_1_Spirochaetes | D_2_Spirochaetia | D_3_Spirochaetales | D_4_Spirochaetaceae | D_5_Sphaerochaeta |
| OTU_3071 | D_1_Firmicutes | D_2_Clostridia | D_3_Clostridiales | D_4_Ruminococcaceae | D_5_Intestinimonas |
| OTU_2250 | D_1_Firmicutes | D_2_Clostridia | D_3_Clostridiales | D_4_Ruminococcaceae | D_5_Pygmaiobacter |
| OTU_661 | D_1_Bacteroidetes | D_2_Bacteroidia | D_3_Bacteroidales |  |  |
| OTU_359 | D_1_Bacteroidetes | D_2_Bacteroidia | D_3_Bacteroidales | D_4_Muribaculaceae |  |
| OTU_674 | D_1_Firmicutes | D_2_Clostridia | D_3_Clostridiales | D_4_Christensenellaceae | D_5_Christensenellaceae R-7 group |
| OTU_504 | D_1_Firmicutes | D_2_Clostridia | D_3_Clostridiales | D_4_Lachnospiraceae |  |
| OTU_526 | D_1_Bacteroidetes | D_2_Bacteroidia | D_3_Bacteroidales | D_4_Rikenellaceae | D_5_Rikenellaceae RC9 gut group |
| OTU_405 | D_1_Firmicutes | D_2_Clostridia | D_3_Clostridiales | D_4_Lachnospiraceae | D_5_[Eubacterium] hallii group |
| OTU_102 | D_1_Proteobacteria | D_2_Deltaproteobacteria | D_3_Desulfovibrionales | D_4_Desulfovibrionaceae | D_5_Desulfovibrio |
| OTU_532 | D_1_Actinobacteria | D_2_Coriobacteriia | D_3_Coriobacteriales | D_4_Coriobacteriaceae | D_5_Enorma |
| OTU_1913 | D_1_Firmicutes | D_2_Clostridia | D_3_Clostridiales | D_4_Lachnospiraceae | D_5_Blautia |
| OTU_792 | D_1_Bacteroidetes | D_2_Bacteroidia | D_3_Bacteroidales | D_4_Rikenellaceae | D_5_Alistipes |
| OTU_315 | D_1_Bacteroidetes | D_2_Bacteroidia | D_3_Bacteroidales | D_4_Bacteroidaceae | D_5_Bacteroides |
| OTU_261 | D_1_Firmicutes | D_2_Clostridia | D_3_Clostridiales | D_4_Christensenellaceae | D_5_Christensenellaceae R-7 group |
| OTU_1324 | D_1_Firmicutes | D_2_Clostridia | D_3_Clostridiales | D_4_Lachnospiraceae | D_5_Blautia |
| OTU_1129 | D_1_Firmicutes | D_2_Clostridia | D_3_Clostridiales | D_4_Ruminococcaceae | D_5_Butyricicoccus |
| OTU_647 | D_1_Bacteroidetes | D_2_Bacteroidia | D_3_Bacteroidales | D_4_Barnesiellaceae | D_5_Barnesiella |
| OTU_223 | D_1_Firmicutes | D_2_Clostridia | D_3_Clostridiales | D_4_Ruminococcaceae | D_5_Ruminococcaceae NK4A214 group |
| OTU_226 | D_1_Bacteroidetes | D_2_Bacteroidia | D_3_Bacteroidales | D_4_Bacteroidaceae | D_5_Bacteroides |
| OTU_758 | D_1_Firmicutes | D_2_Clostridia | D_3_Clostridiales | D_4_Clostridiales vadinBB60 group | D_5_uncultured bacterium |
| OTU_1082 | D_1_Firmicutes | D_2_Clostridia | D_3_Clostridiales | D_4_Peptococcaceae | D_5_Peptococcus |
| OTU_697 | D_1_Actinobacteria | D_2_Coriobacteriia | D_3_Coriobacteriales | D_4_Eggerthellaceae | D_5_Slackia |
| OTU_331 | D_1_Firmicutes | D_2_Clostridia | D_3_Clostridiales | D_4_Ruminococcaceae |  |
| OTU_510 | D_1_Firmicutes | D_2_Clostridia | D_3_Clostridiales | D_4_Clostridiales vadinBB60 group | Ambiguous_taxa |
| OTU_668 | D_1_Bacteroidetes | D_2_Bacteroidia | D_3_Bacteroidales | D_4_Rikenellaceae | D_5_Alistipes |
| OTU_200 | D_1_Bacteroidetes | D_2_Bacteroidia | D_3_Bacteroidales | D_4_Bacteroidaceae | D_5_Bacteroides |
| OTU_196 | D_1_Actinobacteria | D_2_Actinobacteria | D_3_Corynebacteriales | D_4_Corynebacteriaceae | D_5_Corynebacterium 1 |
| OTU_547 | D_1_Firmicutes | D_2_Bacilli | D_3_Lactobacillales | D_4_Aerococcaceae | D_5_Aerococcus |
| OTU_160 | D_1_Firmicutes | D_2_Erysipelotrichia | D_3_Erysipelotrichales | D_4_Erysipelotrichaceae | D_5_Erysipelotrichaceae UCG-003 |
| OTU_855 | D_1_Firmicutes | D_2_Clostridia | D_3_Clostridiales | D_4_Ruminococcaceae | D_5_Ruminococcus 2 |
| OTU_342 | D_1_Firmicutes | D_2_Clostridia | D_3_Clostridiales | D_4_Ruminococcaceae | D_5_Ruminococcaceae UCG-013 |
| OTU_384 | D_1_Bacteroidetes | D_2_Bacteroidia | D_3_Bacteroidales |  |  |
| OTU_206 | D_1_Bacteroidetes | D_2_Bacteroidia | D_3_Bacteroidales | D_4_Prevotellaceae | D_5_Prevotellaceae NK3B31 group |
| OTU_1547 | D_1_Firmicutes | D_2_Bacilli | D_3_Lactobacillales | D_4_Carnobacteriaceae | D_5_Jeotgalibaca |
| OTU_153 | D_1_Firmicutes | D_2_Bacilli | D_3_Lactobacillales | D_4_Aerococcaceae | D_5_Facklamia |
| OTU_175 | D_1_Firmicutes | D_2_Clostridia | D_3_Clostridiales | D_4_Clostridiales vadinBB60 group | D_5_uncultured bacterium |
| OTU_632 | D_1_Firmicutes | D_2_Clostridia | D_3_Clostridiales | D_4_Clostridiales vadinBB60 group | D_5_uncultured bacterium |
| OTU_220 | D_1_Firmicutes | D_2_Negativicutes | D_3_Selenomonadales | D_4_Veillonellaceae | D_5_Veillonella |
| OTU_275 | D_1_Bacteroidetes | D_2_Bacteroidia | D_3_Bacteroidales | D_4_Muribaculaceae | D_5_uncultured bacterium |
| **SC-b** |  |  |  |  |  |
| OTU_229 | D_1_Firmicutes | D_2_Clostridia | D_3_Clostridiales | D_4_Lachnospiraceae | D_5_Tyzzerella 3 |
| OTU_2396 | D_1_Firmicutes | D_2_Clostridia | D_3_Clostridiales | D_4_Ruminococcaceae | D_5_Faecalibacterium |
| OTU_193 | D_1_Bacteroidetes | D_2_Bacteroidia | D_3_Bacteroidales |  |  |
| OTU_323 | D_1_Firmicutes | D_2_Erysipelotrichia | D_3_Erysipelotrichales | D_4_Erysipelotrichaceae | D_5_Erysipelotrichaceae UCG-004 |
| OTU_622 | D_1_Firmicutes | D_2_Erysipelotrichia | D_3_Erysipelotrichales | D_4_Erysipelotrichaceae | D_5_Erysipelotrichaceae UCG-003 |
| OTU_458 | D_1_Firmicutes | D_2_Clostridia | D_3_Clostridiales | D_4_Ruminococcaceae | D_5_Ruminococcaceae UCG-013 |
| OTU_409 | D_1_Firmicutes | D_2_Clostridia | D_3_Clostridiales | D_4_Ruminococcaceae | D_5_Ruminococcus 1 |
| OTU_124 | D_1_Firmicutes | D_2_Clostridia | D_3_Clostridiales | D_4_Ruminococcaceae | D_5_Ruminococcus 2 |
| OTU_170 | D_1_Firmicutes | D_2_Clostridia | D_3_Clostridiales | D_4_Ruminococcaceae | D_5_Ruminococcaceae UCG-014 |
| OTU_137 | D_1_Firmicutes | D_2_Clostridia | D_3_Clostridiales | D_4_Ruminococcaceae | D_5_Ruminococcaceae UCG-014 |
| OTU_239 | D_1_Firmicutes | D_2_Clostridia | D_3_Clostridiales | D_4_Ruminococcaceae | D_5_[Eubacterium] coprostanoligenes group |
| OTU_560 | D_1_Proteobacteria | D_2_Gammaproteobacteria | D_3_Betaproteobacteriales | D_4_Burkholderiaceae | D_5_Parasutterella |
| OTU_69 | D_1_Bacteroidetes | D_2_Bacteroidia | D_3_Bacteroidales | D_4_Barnesiellaceae |  |
| OTU_208 | D_1_Firmicutes | D_2_Clostridia | D_3_Clostridiales | D_4_Ruminococcaceae | D_5_Ruminococcus 1 |
| OTU_78 | D_1_Firmicutes | D_2_Clostridia | D_3_Clostridiales | D_4_Ruminococcaceae | D_5_Ruminococcaceae UCG-005 |
| OTU_50 | D_1_Bacteroidetes | D_2_Bacteroidia | D_3_Bacteroidales | D_4_Prevotellaceae |  |
| OTU_42 | D_1_Spirochaetes | D_2_Spirochaetia | D_3_Spirochaetales | D_4_Spirochaetaceae | D_5_Treponema 2 |
| OTU_167 | D_1_Bacteroidetes | D_2_Bacteroidia | D_3_Bacteroidales | D_4_Prevotellaceae |  |
| OTU_31 | D_1_Bacteroidetes | D_2_Bacteroidia | D_3_Bacteroidales | D_4_Rikenellaceae | D_5_Rikenellaceae RC9 gut group |
| OTU_129 | D_1_Firmicutes | D_2_Clostridia | D_3_Clostridiales | D_4_Ruminococcaceae | D_5_Ruminococcaceae UCG-014 |
| OTU_289 | D_1_Bacteroidetes | D_2_Bacteroidia | D_3_Bacteroidales | D_4_Prevotellaceae | D_5_Prevotellaceae UCG-001 |
| OTU_59 | D_1_Firmicutes | D_2_Clostridia | D_3_Clostridiales | D_4_Ruminococcaceae | D_5_Ruminococcus 1 |
| OTU_602 | D_1_Proteobacteria | D_2_Deltaproteobacteria | D_3_Desulfovibrionales | D_4_Desulfovibrionaceae | D_5_Desulfovibrio |
| OTU_1448 | D_1_Firmicutes | D_2_Clostridia | D_3_Clostridiales | D_4_Ruminococcaceae | D_5_Ruminococcaceae UCG-005 |
| OTU_138 | D_1_Bacteroidetes | D_2_Bacteroidia | D_3_Bacteroidales |  |  |
| OTU_332 | D_1_Bacteroidetes | D_2_Bacteroidia | D_3_Bacteroidales | D_4_Muribaculaceae | D_5_uncultured bacterium |
| OTU_218 | D_1_Firmicutes | D_2_Clostridia | D_3_Clostridiales | D_4_Ruminococcaceae | D_5_Ruminococcaceae UCG-014 |
| OTU_169 | D_1_Firmicutes | D_2_Clostridia | D_3_Clostridiales | D_4_Christensenellaceae | D_5_Christensenellaceae R-7 group |
| OTU_147 | D_1_Bacteroidetes | D_2_Bacteroidia | D_3_Bacteroidales | D_4_Prevotellaceae | D_5_Prevotellaceae UCG-001 |
| OTU_291 | D_1_Bacteroidetes | D_2_Bacteroidia | D_3_Bacteroidales | D_4_Prevotellaceae | D_5_Prevotellaceae UCG-001 |
| OTU_88 | D_1_Firmicutes | D_2_Clostridia | D_3_Clostridiales | D_4_Ruminococcaceae | D_5_Faecalibacterium |
| OTU_316 | D_1_Firmicutes | D_2_Clostridia | D_3_Clostridiales | D_4_Ruminococcaceae | D_5_Ruminiclostridium 6 |
| OTU_145 | D_1_Spirochaetes | D_2_Spirochaetia | D_3_Spirochaetales | D_4_Spirochaetaceae | D_5_Sphaerochaeta |
| OTU_656 | D_1_Bacteroidetes | D_2_Bacteroidia | D_3_Bacteroidales | D_4_Rikenellaceae | D_5_Rikenellaceae RC9 gut group |
| OTU_204 | D_1_Bacteroidetes | D_2_Bacteroidia | D_3_Bacteroidales | D_4_Bacteroidaceae | D_5_Bacteroides |
| OTU_255 | D_1_Firmicutes | D_2_Clostridia | D_3_Clostridiales | D_4_Ruminococcaceae | D_5_Ruminococcaceae UCG-014 |
| OTU_225 | D_1_Bacteroidetes | D_2_Bacteroidia | D_3_Bacteroidales | D_4_Rikenellaceae | D_5_Rikenellaceae RC9 gut group |
| **SC-c** |  |  |  |  |  |
| OTU_83 | D_1_Firmicutes | D_2_Clostridia | D_3_Clostridiales | D_4_Ruminococcaceae | D_5_Ruminococcaceae UCG-002 |
| OTU_617 | D_1_Firmicutes | D_2_Clostridia | D_3_Clostridiales | D_4_Ruminococcaceae | D_5_Ruminococcaceae UCG-002 |
| OTU_247 | D_1_Firmicutes | D_2_Clostridia | D_3_Clostridiales | D_4_Lachnospiraceae | D_5_Lachnospiraceae AC2044 group |
| OTU_424 | D_1_Firmicutes | D_2_Clostridia | D_3_Clostridiales | D_4_Lachnospiraceae | D_5_Lachnospiraceae NK4A136 group |
| OTU_3847 | D_1_Firmicutes | D_2_Clostridia | D_3_Clostridiales | D_4_Lachnospiraceae | D_5_Lachnospiraceae NK4A136 group |
| OTU_2699 | D_1_Firmicutes | D_2_Clostridia | D_3_Clostridiales | D_4_Lachnospiraceae | D_5_Coprococcus 3 |
| OTU_236 | D_1_Bacteroidetes | D_2_Bacteroidia | D_3_Bacteroidales | D_4_Bacteroidaceae | D_5_Bacteroides |
| OTU_192 | D_1_Firmicutes | D_2_Clostridia | D_3_Clostridiales | D_4_Ruminococcaceae | D_5_Ruminococcaceae UCG-014 |
| OTU_172 | D_1_Firmicutes | D_2_Clostridia | D_3_Clostridiales | D_4_Ruminococcaceae | D_5_[Eubacterium] coprostanoligenes group |
| OTU_778 | D_1_Firmicutes | D_2_Clostridia | D_3_Clostridiales | D_4_Ruminococcaceae | D_5_Ruminococcaceae NK4A214 group |
| OTU_77 | D_1_Firmicutes | D_2_Clostridia | D_3_Clostridiales | D_4_Lachnospiraceae | D_5_Lachnospiraceae AC2044 group |
| OTU_101 | D_1_Firmicutes | D_2_Clostridia | D_3_Clostridiales | D_4_Ruminococcaceae | D_5_Ruminococcus 1 |
| OTU_150 | D_1_Bacteroidetes | D_2_Bacteroidia | D_3_Bacteroidales | D_4_Rikenellaceae | D_5_Rikenellaceae RC9 gut group |
| OTU_840 | D_1_Firmicutes | D_2_Clostridia | D_3_Clostridiales | D_4_Christensenellaceae | D_5_Christensenellaceae R-7 group |
| OTU_187 | D_1_Firmicutes | D_2_Clostridia | D_3_Clostridiales | D_4_Lachnospiraceae | D_5_Lachnospiraceae UCG-008 |
| OTU_353 | D_1_Firmicutes | D_2_Clostridia | D_3_Clostridiales | D_4_Lachnospiraceae | D_5_Blautia |
| OTU_28 | D_1_Firmicutes | D_2_Clostridia | D_3_Clostridiales | D_4_Lachnospiraceae | D_5_Lachnospiraceae UCG-009 |
| OTU_486 | D_1_Firmicutes | D_2_Clostridia | D_3_Clostridiales | D_4_Ruminococcaceae | D_5_Ruminococcus 1 |
| OTU_68 | D_1_Bacteroidetes | D_2_Bacteroidia | D_3_Bacteroidales | D_4_M2PB4-65 termite group | D_5_uncultured bacterium |
| OTU_413 | D_1_Firmicutes | D_2_Clostridia | D_3_Clostridiales | D_4_Ruminococcaceae | D_5_Oscillibacter |
| OTU_292 | D_1_Bacteroidetes | D_2_Bacteroidia | D_3_Bacteroidales | D_4_Rikenellaceae | D_5_Rikenellaceae RC9 gut group |
| OTU_1051 | D_1_Firmicutes | D_2_Clostridia | D_3_Clostridiales | D_4_Christensenellaceae | D_5_Christensenellaceae R-7 group |
| OTU_283 | D_1_Firmicutes | D_2_Clostridia | D_3_Clostridiales | D_4_Family XIII | D_5_Family XIII AD3011 group |
| OTU_182 | D_1_Firmicutes | D_2_Clostridia | D_3_Clostridiales | D_4_Christensenellaceae | D_5_Christensenellaceae R-7 group |
| OTU_161 | D_1_Firmicutes | D_2_Clostridia | D_3_Clostridiales | D_4_Christensenellaceae | D_5_Christensenellaceae R-7 group |
| OTU_403 | D_1_Firmicutes | D_2_Clostridia | D_3_Clostridiales | D_4_Family XIII | D_5_Family XIII AD3011 group |
| OTU_845 | D_1_Firmicutes | D_2_Clostridia | D_3_Clostridiales | D_4_Christensenellaceae | D_5_Christensenellaceae R-7 group |
| OTU_216 | D_1_Firmicutes | D_2_Clostridia | D_3_Clostridiales | D_4_Lachnospiraceae | D_5_Lachnoclostridium 10 |
| OTU_416 | D_1_Firmicutes | D_2_Clostridia | D_3_Clostridiales | D_4_Ruminococcaceae | D_5_[Eubacterium] coprostanoligenes group |
| OTU_633 | D_1_Firmicutes | D_2_Clostridia | D_3_Clostridiales | D_4_Ruminococcaceae | D_5_Ruminococcus 1 |
| OTU_716 | D_1_Spirochaetes | D_2_Spirochaetia | D_3_Spirochaetales | D_4_Spirochaetaceae | D_5_Treponema 2 |
| **SC-d** |  |  |  |  |  |
| OTU_328 | D_1_Proteobacteria | D_2_Gammaproteobacteria | D_3_Betaproteobacteriales | D_4_Burkholderiaceae | D_5_Hydrogenophaga |
| OTU_64 | D_1_Proteobacteria | D_2_Gammaproteobacteria | D_3_Pseudomonadales | D_4_Pseudomonadaceae | D_5_Pseudomonas |
| OTU_95 | D_1_Proteobacteria | D_2_Gammaproteobacteria | D_3_Betaproteobacteriales | D_4_Burkholderiaceae | D_5_Herbaspirillum |
| OTU_142 | D_1_Proteobacteria | D_2_Gammaproteobacteria | D_3_Betaproteobacteriales | D_4_Burkholderiaceae | D_5_Ralstonia |
| OTU_105 | D_1_Proteobacteria | D_2_Alphaproteobacteria | D_3_Caulobacterales | D_4_Caulobacteraceae | D_5_Brevundimonas |
| OTU_2138 | D_1_Proteobacteria | D_2_Alphaproteobacteria | D_3_Sphingomonadales | D_4_Sphingomonadaceae | D_5_Croceicoccus |
| OTU_109 | D_1_Proteobacteria | D_2_Gammaproteobacteria | D_3_Betaproteobacteriales | D_4_Burkholderiaceae | D_5_Cupriavidus |
| OTU_319 | D_1_Acidobacteria | D_2_Holophagae | D_3_Subgroup 7 | D_4_uncultured bacterium | D_5_uncultured bacterium |
| OTU_217 | D_1_Proteobacteria | D_2_Gammaproteobacteria | D_3_Betaproteobacteriales | D_4_Burkholderiaceae | D_5_Pelomonas |
| OTU_44 | D_1_Proteobacteria | D_2_Gammaproteobacteria | D_3_Betaproteobacteriales | D_4_Burkholderiaceae | D_5_Burkholderia-Caballeronia-Paraburkholderia |
| OTU_721 | D_1_Proteobacteria | D_2_Gammaproteobacteria | D_3_Betaproteobacteriales | D_4_Burkholderiaceae | D_5_Acidovorax |
| OTU_130 | D_1_Acidobacteria | D_2_Subgroup 6 | D_3_uncultured Acidobacterium sp. | D_4_uncultured Acidobacterium sp. | D_5_uncultured Acidobacterium sp. |
| OTU_98 | D_1_Proteobacteria | D_2_Gammaproteobacteria | D_3_Pseudomonadales | D_4_Moraxellaceae | D_5_Acinetobacter |
| OTU_140 | D_1_Proteobacteria | D_2_Gammaproteobacteria | D_3_Betaproteobacteriales | D_4_Rhodocyclaceae | D_5_Methyloversatilis |
| OTU_453 | D_1_Proteobacteria | D_2_Gammaproteobacteria | D_3_Pseudomonadales | D_4_Moraxellaceae | D_5_Acinetobacter |
| OTU_344 | D_1_Proteobacteria | D_2_Gammaproteobacteria | D_3_Betaproteobacteriales | D_4_Rhodocyclaceae | D_5_Dechloromonas |
| OTU_174 | D_1_Actinobacteria | D_2_Actinobacteria | D_3_Propionibacteriales | D_4_Nocardioidaceae | D_5_Aeromicrobium |
| OTU_608 | D_1_Proteobacteria | D_2_Alphaproteobacteria | D_3_Caulobacterales | D_4_Caulobacteraceae | D_5_Brevundimonas |
| OTU_2333 | D_1_Proteobacteria | D_2_Alphaproteobacteria | D_3_Sphingomonadales | D_4_Sphingomonadaceae | D_5_Sphingomonas |
| OTU_399 | D_1_Bacteroidetes | D_2_Bacteroidia | D_3_Chitinophagales | D_4_Chitinophagaceae | D_5_Flavisolibacter |
| OTU_616 | D_1_Proteobacteria | D_2_Alphaproteobacteria | D_3_Sphingomonadales | D_4_Sphingomonadaceae | D_5_Sphingopyxis |
| **SC-e** |  |  |  |  |  |
| OTU_3332 | D_1_Proteobacteria | D_2_Alphaproteobacteria | D_3_Rhizobiales | D_4_Rhizobiaceae | D_5_Mesorhizobium |
| OTU_205 | D_1_Proteobacteria | D_2_Gammaproteobacteria | D_3_Cellvibrionales | D_4_Cellvibrionaceae | D_5_Cellvibrio |
| OTU_1198 | D_1_Proteobacteria | D_2_Alphaproteobacteria | D_3_Acetobacterales | D_4_Acetobacteraceae |  |
| OTU_599 | D_1_Proteobacteria | D_2_Alphaproteobacteria | D_3_Caulobacterales | D_4_Caulobacteraceae | D_5_uncultured |
| OTU_587 | D_1_Actinobacteria | D_2_Actinobacteria | D_3_Micrococcales | D_4_Intrasporangiaceae |  |
| OTU_1267 | D_1_Actinobacteria | D_2_Actinobacteria | D_3_Propionibacteriales | D_4_Nocardioidaceae | D_5_Marmoricola |
| OTU_386 | D_1_Actinobacteria | D_2_Actinobacteria | D_3_Micrococcales | D_4_Dermacoccaceae | D_5_Branchiibius |
| OTU_1060 | D_1_Actinobacteria | D_2_Actinobacteria | D_3_Corynebacteriales | D_4_Mycobacteriaceae | D_5_Mycobacterium |
| OTU_1278 | D_1_Actinobacteria | D_2_Thermoleophilia | D_3_Gaiellales | D_4_uncultured | Ambiguous_taxa |
| OTU_122 | D_1_Proteobacteria | D_2_Gammaproteobacteria | D_3_Xanthomonadales | D_4_Rhodanobacteraceae | D_5_Rhodanobacter |
| OTU_1421 | D_1_Proteobacteria | D_2_Gammaproteobacteria | D_3_Betaproteobacteriales | D_4_SC-I-84 | D_5_uncultured beta proteobacterium |
| OTU_1092 | D_1_Proteobacteria | D_2_Alphaproteobacteria | D_3_Rhizobiales | D_4_Xanthobacteraceae | D_5_Bradyrhizobium |
| OTU_1293 | D_1_Nitrospirae | D_2_Nitrospira | D_3_Nitrospirales | D_4_Nitrospiraceae | D_5_Nitrospira |
| OTU_1665 | D_1_Proteobacteria | D_2_Alphaproteobacteria | D_3_Rhizobiales | D_4_Xanthobacteraceae | D_5_uncultured |
| OTU_1338 | D_1_Proteobacteria | D_2_Alphaproteobacteria | D_3_Sphingomonadales | D_4_Sphingomonadaceae | D_5_Sphingomonas |
| OTU_769 | D_1_Chloroflexi | D_2_KD4-96 | D_3_uncultured bacterium | D_4_uncultured bacterium | D_5_uncultured bacterium |
| OTU_2234 | D_1_Acidobacteria | D_2_Blastocatellia (Subgroup 4) | D_3_Blastocatellales | D_4_Blastocatellaceae | D_5_JGI 0001001-H03 |
| OTU_2486 | D_1_Actinobacteria | D_2_Actinobacteria | D_3_Micrococcales | D_4_Intrasporangiaceae |  |
| OTU_410 | D_1_Chloroflexi | D_2_KD4-96 | D_3_uncultured bacterium | D_4_uncultured bacterium | D_5_uncultured bacterium |
| OTU_1006 | D_1_Proteobacteria | D_2_Deltaproteobacteria | D_3_Myxococcales | D_4_Haliangiaceae | D_5_Haliangium |
| OTU_1937 | D_1_Actinobacteria | D_2_Thermoleophilia | D_3_Gaiellales | D_4_uncultured |  |
| OTU_1313 | D_1_Patescibacteria | D_2_Saccharimonadia | D_3_Saccharimonadales | D_4_uncultured bacterium | D_5_uncultured bacterium |
| OTU_295 | D_1_Proteobacteria | D_2_Gammaproteobacteria | D_3_Betaproteobacteriales | D_4_SC-I-84 | D_5_uncultured bacterium |
| OTU_1379 | D_1_Proteobacteria | D_2_Gammaproteobacteria | D_3_Betaproteobacteriales | D_4_Burkholderiaceae | D_5_Paucibacter |
| OTU_839 | D_1_Actinobacteria | D_2_Actinobacteria | D_3_Propionibacteriales | D_4_Nocardioidaceae | D_5_Nocardioides |
| OTU_468 | D_1_Proteobacteria | D_2_Gammaproteobacteria | D_3_Xanthomonadales | D_4_Rhodanobacteraceae | D_5_uncultured |
| OTU_922 | D_1_Actinobacteria | D_2_Actinobacteria | D_3_Micrococcales | D_4_Microbacteriaceae | D_5_Humibacter |
| OTU_471 | D_1_Actinobacteria | D_2_Actinobacteria | D_3_Micrococcales | D_4_Micrococcaceae | D_5_Sinomonas |
| OTU_346 | D_1_Actinobacteria | D_2_Actinobacteria | D_3_Micrococcales | D_4_Intrasporangiaceae |  |
| OTU_1567 | D_1_Planctomycetes | D_2_Planctomycetacia | D_3_Gemmatales | D_4_Gemmataceae | D_5_uncultured |
| OTU_906 | D_1_Proteobacteria | D_2_Gammaproteobacteria | D_3_KF-JG30-C25 | D_4_uncultured proteobacterium | D_5_uncultured proteobacterium |
| OTU_731 | D_1_Proteobacteria | D_2_Gammaproteobacteria | D_3_Xanthomonadales | D_4_Xanthomonadaceae | D_5_Lysobacter |
| OTU_1799 | D_1_Chloroflexi | D_2_Ktedonobacteria | D_3_Ktedonobacterales | D_4_JG30-KF-AS9 | D_5_uncultured bacterium |
| OTU_1500 | D_1_Proteobacteria | D_2_Gammaproteobacteria | D_3_Betaproteobacteriales | D_4_SC-I-84 |  |
| OTU_1350 | D_1_Chloroflexi | D_2_JG30-KF-CM66 | D_3_uncultured bacterium | D_4_uncultured bacterium | D_5_uncultured bacterium |
| OTU_369 | D_1_Firmicutes | D_2_Bacilli | D_3_Bacillales | D_4_Bacillaceae | D_5_Bacillus |
| OTU_1241 | D_1_Proteobacteria | D_2_Gammaproteobacteria | D_3_Betaproteobacteriales | D_4_Nitrosomonadaceae | D_5_Ellin6067 |
| OTU_962 | D_1_Proteobacteria | D_2_Alphaproteobacteria | D_3_Rhizobiales | D_4_Hyphomicrobiaceae | D_5_Hyphomicrobium |
| OTU_1369 | D_1_Proteobacteria | D_2_Gammaproteobacteria | D_3_Betaproteobacteriales | D_4_Burkholderiaceae | D_5_Castellaniella |
| OTU_624 | D_1_Bacteroidetes | D_2_Bacteroidia | D_3_Flavobacteriales | D_4_Weeksellaceae |  |
| **SC-f** |  |  |  |  |  |
| OTU_5 | D_1_Firmicutes | D_2_Bacilli | D_3_Lactobacillales | D_4_Lactobacillaceae | D_5_Lactobacillus |
| OTU_54 | D_1_Firmicutes | D_2_Bacilli | D_3_Lactobacillales | D_4_Lactobacillaceae | D_5_Lactobacillus |
| OTU_32 | D_1_Firmicutes | D_2_Bacilli | D_3_Lactobacillales | D_4_Lactobacillaceae | D_5_Lactobacillus |
| OTU_9 | D_1_Firmicutes | D_2_Bacilli | D_3_Lactobacillales | D_4_Lactobacillaceae | D_5_Lactobacillus |
| OTU_103 | D_1_Firmicutes | D_2_Bacilli | D_3_Lactobacillales | D_4_Lactobacillaceae | D_5_Lactobacillus |
| OTU_51 | D_1_Firmicutes | D_2_Bacilli | D_3_Lactobacillales | D_4_Lactobacillaceae | D_5_Lactobacillus |
| OTU_86 | D_1_Actinobacteria | D_2_Actinobacteria | D_3_Bifidobacteriales | D_4_Bifidobacteriaceae | D_5_Aeriscardovia |
| OTU_520 | D_1_Firmicutes | D_2_Bacilli | D_3_Lactobacillales | D_4_Lactobacillaceae | D_5_Lactobacillus |
| OTU_180 | D_1_Firmicutes | D_2_Bacilli | D_3_Lactobacillales | D_4_Lactobacillaceae | D_5_Lactobacillus |
| OTU_629 | D_1_Firmicutes | D_2_Bacilli | D_3_Lactobacillales | D_4_Lactobacillaceae | D_5_Lactobacillus |
| OTU_56 | D_1_Firmicutes | D_2_Bacilli | D_3_Lactobacillales | D_4_Lactobacillaceae | D_5_Lactobacillus |
| OTU_329 | D_1_Firmicutes | D_2_Bacilli | D_3_Lactobacillales | D_4_Lactobacillaceae | D_5_Lactobacillus |
| OTU_97 | D_1_Actinobacteria | D_2_Actinobacteria | D_3_Bifidobacteriales | D_4_Bifidobacteriaceae | D_5_Bifidobacterium |
| OTU_136 | D_1_Firmicutes | D_2_Bacilli | D_3_Lactobacillales | D_4_Lactobacillaceae | D_5_Lactobacillus |
| OTU_2509 | D_1_Firmicutes | D_2_Bacilli | D_3_Lactobacillales | D_4_Lactobacillaceae | D_5_Lactobacillus |
| OTU_149 | D_1_Firmicutes | D_2_Bacilli | D_3_Lactobacillales | D_4_Lactobacillaceae | D_5_Lactobacillus |
| OTU_391 | D_1_Firmicutes | D_2_Bacilli | D_3_Lactobacillales | D_4_Lactobacillaceae | D_5_Lactobacillus |
