# Supplementary material for: Covariation of the Fecal Microbiome with Diet in Nonpasserine Birds
Source: mSphere. 2021 May 12;6(3):e00308-21. doi: 10.1128/mSphere.00308-21 (PMC8125056; doi:10.1128/mSphere.00308-21)
Supplement: TABLE S5 [file mSphere.00308-21-st005.docx]

**Table. S5** Comparison of the co-occurrence percent and total abundance of the microbiota communities between the pairwise groups. BH-adjusted p-value<0.05 were colored (red: percent; green: abundance).

| Sub-network | p-value | Corn-soy | | Fish | | Flesh | | Foliage | | Fruit | | Grain | |
| --- | --- | --- | --- | --- | --- | --- | --- | --- | --- | --- | --- | --- | --- |
|  |  | percent | abundance | percent | abundance | percent | abundance | percent | abundance | percent | abundance | percent | abundance |
| a | Fish | 8.00E-05 | 0.00005 | - | - | - | - | - | - | - | - | - | - |
|  | Flesh | 0.0032 | 0.00034 | 0.16723 | 0.41173 | - | - | - | - | - | - | - | - |
|  | Foliage | 0.00145 | 0.000064 | 0.03728 | 0.0076 | 0.50438 | 0.14685 | - | - | - | - | - | - |
|  | Fruit | 2.80E-07 | 3.1E-07 | 1 | 0.56576 | 0.19159 | 0.11022 | 0.01088 | 0.00097 | - | - | - | - |
|  | Grain | 0.00014 | 0.000064 | 0.08304 | 0.01644 | 0.30657 | 0.10579 | 0.6208 | 0.87474 | 0.01256 | 0.00196 | - | - |
|  | Omni | 0.00014 | 0.00005 | 0.00014 | 0.00015 | 0.01272 | 0.00809 | 0.22295 | 0.21471 | 4.20E-08 | 2.1E-08 | 0.08304 | 0.214 |
| b | Fish | 0.0027 | 0.0028 | - | - | - | - | - | - | - | - | - | - |
|  | Flesh | 0.4218 | 0.227 | 0.0505 | 0.0505 | - | - | - | - | - | - | - | - |
|  | Foliage | 0.1673 | 0.2685 | 0.0296 | 0.0342 | 1 | 0.8247 | - | - | - | - | - | - |
|  | Fruit | 3.10E-05 | 2.70E-05 | 0.4218 | 0.437 | 0.0432 | 0.0445 | 0.0094 | 0.0082 | - | - | - | - |
|  | Grain | 0.02 | 0.0445 | 0.1593 | 0.1596 | 0.4218 | 0.5779 | 0.3199 | 0.389 | 0.1673 | 0.1596 | - | - |
|  | Omni | 0.4218 | 0.437 | 0.0019 | 0.0019 | 0.2747 | 0.2137 | 0.119 | 0.1643 | 6.20E-06 | 5.50E-06 | 0.0094 | 0.0173 |
| c | Fish | 0.9 | 0.76 | - | - | - | - | - | - | - | - | - | - |
|  | Flesh | 0.28 | 0.32 | 0.35 | 0.4 | - | - | - | - | - | - | - | - |
|  | Foliage | 0.4 | 0.51 | 0.63 | 0.7 | 0.4 | 0.45 | - | - | - | - | - | - |
|  | Fruit | 0.26 | 0.32 | 0.4 | 0.48 | 0.4 | 0.48 | 0.79 | 0.76 | - | - | - | - |
|  | Grain | 0.25 | 0.32 | 0.32 | 0.45 | 0.57 | 0.64 | 0.47 | 0.64 | 0.57 | 0.7 | - | - |
|  | Omni | 0.9 | 0.76 | 0.79 | 0.67 | 0.28 | 0.32 | 0.4 | 0.48 | 0.25 | 0.32 | 0.25 | 0.32 |
| d | Fish | 0.501 | 0.0012 | - | - | - | - | - | - | - | - | - | - |
|  | Flesh | 0.712 | 0.15392 | 0.501 | 0.35669 | - | - | - | - | - | - | - | - |
|  | Foliage | 0.261 | 0.00072 | 0.734 | 0.91635 | 0.191 | 0.2422 | - | - | - | - | - | - |
|  | Fruit | 0.038 | 4.20E-08 | 0.501 | 0.35669 | 0.038 | 0.05664 | 0.876 | 0.2422 | - | - | - | - |
|  | Grain | 0.963 | 0.00116 | 0.501 | 0.98536 | 0.963 | 0.35669 | 0.191 | 0.91635 | 0.027 | 0.29184 | - | - |
|  | Omni | 0.963 | 0.05664 | 0.501 | 0.05664 | 0.585 | 0.98536 | 0.198 | 0.05664 | 0.015 | 1.10E-05 | 0.963 | 0.05664 |
| e | Fish | 0.0048 | 0.00083 | - | - | - | - | - | - | - | - | - | - |
|  | Flesh | 0.0062 | 0.00447 | 0.3666 | 0.41494 | - | - | - | - | - | - | - | - |
|  | Foliage | 0.0129 | 0.0013 | 0.678 | 0.41707 | 0.3442 | 0.16783 | - | - | - | - | - | - |
|  | Fruit | 0.004 | 0.00082 | 0.6524 | 0.75707 | 0.678 | 0.63027 | 0.3666 | 0.31679 | - | - | - | - |
|  | Grain | 0.6957 | 0.10039 | 0.0062 | 0.00508 | 0.0089 | 0.01198 | 0.0184 | 0.03511 | 0.0048 | 0.00486 | - | - |
|  | Omni | 0.004 | 0.00102 | 0.8795 | 0.41494 | 0.3442 | 0.16783 | 0.678 | 0.83922 | 0.3483 | 0.12379 | 0.006 | 0.03549 |
| f | Fish | 0.00017 | 0.00015 | - | - | - | - | - | - | - | - | - | - |
|  | Flesh | 0.01078 | 0.01217 | 0.51538 | 0.47652 | - | - | - | - | - | - | - | - |
|  | Foliage | 0.01078 | 0.01217 | 0.12392 | 0.09817 | 0.51907 | 0.47652 | - | - | - | - | - | - |
|  | Fruit | 0.01078 | 0.38773 | 0.00013 | 6.70E-05 | 0.03515 | 0.01538 | 0.10513 | 0.02434 | - | - | - | - |
|  | Grain | 0.14985 | 0.00102 | 0.00011 | 4.60E-05 | 0.0028 | 0.00321 | 0.00107 | 0.00028 | 0.00011 | 4.60E-05 | - | - |
|  | Omni | 0.0028 | 0.0041 | 0.00221 | 0.00111 | 0.14563 | 0.1394 | 0.37036 | 0.37296 | 0.04767 | 0.01172 | 0.00011 | 3.00E-06 |
